# Potential harms of social prescribing: a global umbrella review and dark logic model

**DOI:** 10.1136/bmjopen-2025-108998

**Published:** 2026-05-04

**Authors:** Matthew Cooper, Daniel Okeowo, Liesel Bennett, Eman Aslam, Faiza Yahya, Lauren Lawson, Anna Robinson-Barella, Hamde Nazar, Jason Scott

**Affiliations:** 1 Newcastle Patient Safety Research Collaboration, Newcastle University, Newcastle upon Tyne, Tyne and Wear, UK; 2 School of Psychology, Newcastle University, Newcastle upon Tyne, Tyne and Wear, UK; 3 School of Pharmacy, Newcastle University, Newcastle upon Tyne, Tyne and Wear, UK; 4 Faculty of Health and Life Sciences, Northumbria University, Newcastle upon Tyne, UK

**Keywords:** SOCIAL MEDICINE, General Practice, PUBLIC HEALTH

## Abstract

**Abstract:**

**Objectives:**

Social prescribing has provided a lifeline to people, capturing the importance of quality support offered via community connections. It has not been exempt from difficulties, resulting in low-quality evidence on effectiveness in part due to high drop-out rates and lack of controls. While these have been the focus of recent research, less attention has been paid to the safety implications of social prescribing. This review aims to understand safety implications of social prescribing interventions and to build a dark logic model of how harms are produced.

**Design:**

Using review of review (umbrella) methodology, we searched nine databases to June 2024.

**Data sources:**

Medline/Ovid, Embase, PsycINFO, Cochrane Library/Wiley, Web of Science and Scopus) were searched from 1 January 2010 to 3 June 2024.

**Eligibility criteria:**

Included reviews were systematic/scoping/narrative reviews or meta-analyses, which synthesised primary data collected from any person in receipt, involved in delivery or the commissioning of social prescribing interventions. The context of the review was social prescribing interventions/services based in primary healthcare (statutory) or third sector (non-statutory) in any country. Only reviews that were published in English and in peer-reviewed journals were included.

**Data extraction and synthesis:**

Two independent reviewers extracted data from included reviews using the Typology of Harms Framework (which includes five categories of harms associated with interventions: physical, psychological, group/social, equity and opportunity harms) and to build a dark logic model to understand what contributes to these harms.

**Results:**

Sixteen reviews were included, reporting on social prescribing research including a link worker. Of the identified harms, we found that opportunity harms (harm related to the cost, inappropriate or ineffective interventions) were most reported. There was also evidence extracted to suggest plausible psychological, equity (impact caused by inequity in provision, delivery or access) and group/social harms (impact that overly or inadvertently excludes a person). No physical harms were identified.

**Conclusions:**

Social prescribing, as with any delivery of care, has the potential to cause harm. We identified a range of potential harms (psychological, group/social, equity or opportunity harm) from social prescribing; however, it is unlikely to be an exhaustive list. We provide two clear outcomes: (1) the need for robust design of social prescribing research, including collection of data on the incidence and prevalence of harms, (2) recognition of the potential for harm from social prescribing and to address these where practicable.

**STRENGTHS AND LIMITATIONS OF THIS STUDY**

- This is the first study to categorise the potential harms associated with social prescribing and how the harms may arise.
- A range of potential harms to patients in receipt of or eligible for social prescribing were identified that related to the organisation and delivery of social prescribing services.
- Providing an evidence-informed plausible harm framework sets a clear need for people involved in the organisation and delivery of social prescribing to address the issues raised and for future research to explore prevention strategies.
- There is a lack of a globally recognised definition of social prescribing, and often no direct translation in many languages, limiting evidence included in this review.
- Identification of harms is notoriously difficult, often requiring multiple methods that each have their limitations.

## Introduction

Social prescribing is a holistic approach to healthcare delivered within the community (such as within neighbourhoods, local areas or estates). There has been a rapid global rollout of social prescribing since its inclusion in the National Health Service (NHS) in England planning,^1^ and is being adopted across many high-income countries such as Australia, Canada and Singapore.^2^ It is globally defined as follows:

a means for trusted individuals in clinical and community settings to identify that a person has non-medical, health-related social needs and to subsequently connect them to non-clinical supports and services within the community by co-producing a social prescription—a non-medical prescription, to improve health and well-being and to strengthen community connections.^3^

Social prescribing is a key example of how health and social care can be integrated within communities to empower patients to take control of their health. Central to this is patient choice around what, where and when they receive support for their individual needs. Enabling patient choice along with social prescribing and community-based support forms two of the six pillars of personalised care within the NHS.^4^ Social prescribing has provided a vital lifeline to people, with research capturing the importance of quality support offered via community connections^5^ and clear evidence that some patients value the support provided by social prescribing services.^5^ Social prescribing is delivered by a link worker, whose role is defined as someone who connects an individual to support and services to improve their health (social, mental and physical) and well-being. Research has previously advocated social prescribing for supporting patients with obesity,^6^ diabetes^7^ and mental ill health,^8^ and it has been shown to have a positive social return on investment (up to £4.70 for every £1 invested)^6^ with estimates suggesting social prescribing can contribute an additional 91.7 quality-adjusted life years.^7^

However, social prescribing has not been exempt from difficulties associated with both developing an evidence base for social prescribing and the delivery of social prescribing interventions. Consistent issues with the design of reviews include regular use of designs that are unable to accurately determine effectiveness, such as pre–post reviews and high drop-out rates among participants.^8^ Research has also widely reported issues with attrition across delivery, a lack of theoretical underpinning and barriers to acceptance within medicine.^8 9^ There have also been challenges to integration within healthcare systems and the shifting of responsibility for care management from a healthcare professional (HCP) to the patient.^10^ A critical aspect affecting the delivery of social prescribing is the lack of sustainable funding, which has been reported as a widespread challenge across research.^11^ The evidence base for social prescribing is building and now includes attempts to address some of these existing challenges.^12 13^ However, the discourse surrounding social prescribing is largely silent on the associated risks and potential harms.

Patient safety should be a critical consideration for any medical intervention or practice, including care delivered in the community.^14^ Evidence indicates that patient safety is the responsibility of all health and social care actors, from the patient and HCP, services and systems to communities and wider society.^15 16^ A key component of developing safer care is the identification of risks and harms associated with interventions, which then enables the development of approaches to reduce harm.^17^ This umbrella review aims to identify evidence of potential harms associated with social prescribing and to build a dark logic model to understand what contributes to these harms. Dark logic models are frameworks that identify potential adverse or unintended consequences of interventions, complementing traditional models focused on positive outcomes. Their application to social prescribing allows for the mapping of risks such as negative outcomes (eg, increased anxiety from pressure to engage), mechanisms (eg, feelings of inadequacy), contextual factors (eg, transport or financial barriers) and feedback loops (eg, repeated disengagement reducing trust in providers). Applying dark logic provides a starting point (visual summary) for the development, commissioning and delivery of social prescribing services to reflect on how harms may arise in a clearly constructed framework.

## Methods

### Study design

This umbrella review adhered to the Preferred Reporting Items for Systematic review and Meta-Analyses (PRISMA) guidelines and published protocol (CRD42024553272).^18^ A PRISMA checklist for this review is attached in online supplemental Table 1. For access to the search strategy, please see published protocol. Umbrella review methodology was necessary to investigate this research question as there are multiple systematic reviews and meta-analyses of social prescribing, and an umbrella review enables the synthesis of high-level evidence across diverse interventions exposure. Additionally, there has been no single systematic review that has captured the potential harms of social prescribing; instead, it made comments towards aspects of patient harm. Therefore, the volume of data required to answer the question is limited to those reviews that have extracted this data. Umbrella review methodology enhances efficiency by leveraging existing systematic reviews rather than duplicating efforts while following rigorous evidence synthesis guidelines to set future research priorities. This review followed the Methodology for JBI Umbrella Reviews where appropriate.^19^

### Review criteria

Included reviews were systematic/scoping/narrative reviews or meta-analyses, which synthesised peer-reviewed primary data collected from any person in receipt, involved in delivery or the commissioning of social prescribing interventions (defined previously). The context of the review was social prescribing interventions/services based in primary healthcare (statutory) or third sector (non-statutory) in any country. Only reviews that were published in English and in peer-reviewed journals were included. There was no requirement for reviews to present or synthesise outcomes of harm within their results. There were no other restrictions placed on review type (qualitative, quantitative or mixed method) or funder. Reviews were excluded if they included data collected from community-based support that was not part of social prescribing interventions or where the social prescribing intervention did not involve a link worker (or iterations of this title such as community connector, community navigator or community links worker). There were no comparator or control group restrictions. The PIO is stated as follows:

*Population*: any person in receipt (patient, public, community member), involved in delivery (link worker or other title) or commissioning of social prescribing services (funders, governments)

*Intervention*: social prescribing interventions/services (defined previously) based in primary healthcare (statutory care or first point of contact within a healthcare system) or third sector (non-statutory or non-governmental/profit/charity/voluntary) in any country.

*Outcome*: any physical, mental or social measure using qualitative, quantitative or mixed methods at an individual level.

### Search strategy

Nine databases (Medline/Ovid, Embase, PsycINFO, Cochrane Library/Wiley, Web of Science and Scopus) were searched from 1 January 2010 (using analysis data from Web of Science and Scopus to track research publication trends for ‘social prescribing’) to 3 June 2024. Before database searching, pilot searches were conducted to develop the search string and reviewers used their existing expertise in social prescribing to identify key papers to test the search string. The search strategy is available for review in the published protocol.^18^

### Study selection

All database results were uploaded to Rayyan and underwent a process of deduplication by one reviewer (MC). Two reviewers (LB/EA) screened all titles and abstracts independently against the screening criteria. All reviews retained following the screening of titles and abstracts were reassessed in full text by the same two reviewers (LB/EA) independently. Disagreements at each stage were resolved by discussion with a third reviewer (MC/DO). Forwards and backwards citation and reference searching were conducted on all included reviews using Google Scholar.

### Data extraction

All data were extracted using Microsoft Excel by hand, with custom data headings to collect data on review characteristics (country of origin, aims, design, synthesis method, sample size, comorbidities, included references and reported outcomes) as per protocol. Data were extracted from included reviews using the Typology of Harms Framework (see table 1 for definitions and social prescribing application).^20^ The framework was used to provide structure and guidance to the data extraction. It includes five categories of adverse effects of interventions: (1) direct (physical harm), (2) psychological (impact on mental or emotional state), (3) equity (harm associated with widening of inequity), (4) group and social (singling out of people or groups or promoting negative behaviours) and (5) opportunity cost (the loss of potential benefits due to insufficient resource allocation).^20^

**Table 1:** Types of harm and definitions used in this review, adapted Lorenc *et al*^20^

Types of harm	Definition	Application to social prescribing
Physical harm	Any potential negative physical impact as a consequence of social prescribing	Any adverse physical outcomes linked to prescribed activities (eg, injury, health deterioration).
Psychological harm	Any potential negative psychological impact as a result of social prescribing	Emotional distress, increased anxiety or feelings of isolation caused by participation.
Equity harm	Any potential negative structural or systemic inequities in the design, provision or delivery models of social prescribing that result in unequal access, quality of provision or benefits specific groups based on characteristics (age, sex, gender, disability, culture)	Patterned disparities across population characteristics caused by institutional processes/policy, allocation of resources, design of services, evidence used in decision making or organisational practices that systematically advantage some populations over others.
Group/social harm	Any potential negative impact of interpersonal or community-level processes or beliefs (stigma, social stereotypes, scepticism or fear of certain people) that affect a person(s) ability to engage meaningfully with social prescribing or supporting organisation.	Harms caused by social dynamics (stigma, exclusionary behaviour, identity-based exclusion for example) that are engaged to cause disruption to relationships, discomfort, discouragement or discontinued engagement in support.
Opportunity harm	Any potential negative impact which relates to the cost of participation in an intervention, the inadequate or inappropriate provision of resources or the provision of ineffective interventions	Resource misallocation, costs of participation or diversion from more effective alternatives.

### Quality appraisal

To appraise the quality of the included reviews, we used the AMSTAR 2 tool for systematic reviews.^21^ This tool was chosen due to the plausible range of approaches to evidence synthesis employed. The AMSTAR 2 tool is a 16-question checklist that provides structured guidance for the appraiser based on all aspects of a review, from planning to reporting.^21^ Two reviewers (MC and DO) independently completed the AMSTAR 2 tool for each review. Conflicts were resolved through discussion and with the inclusion of a third reviewer where necessary (JS/LB/EA). Within the AMSTAR 2 checklist, questions 11 and 12 (questions around meta-analysis) were not applicable as none of the included reviews were meta-analyses. Items 1–10, 13–16 were applied to all reviews. A maximum score of 14 was achievable (yes=1, no=0). Two reviewers (MC and JS) were excluded from the assessment of one review due to being authors listed.

### Data synthesis

We conducted a narrative synthesis of extracted data to explore each category from the Typology of Harms,^20^ with ‘direct harm’ adapted to ‘physical harm’ to avoid confusion with proximal (direct consequence of social prescribing) and distal causes (indirect consequence of social prescribing), which were also coded. Data were coded by one reviewer (MC) and verified independently by another (JS/DO). To ensure rigour within coding, the definition of harms was defined prior to coding by the review team for consistency of understanding and application. Coding was transparent and completed by two reviewers. We synthesised review characteristics using descriptive statistics and produced a summary of the main patterns and conclusions drawn across reviews. Patterns were reviewed both within and across reviews, with final evidence to support each category of harm reported. In the protocol, it was stated that data would be coded against either patient or healthcare system harm. However, this was not possible due to the limited data (quality and quantity) for healthcare system outcomes. To assess the potential for overlap of reported data between included reviews, the GROOVE tool was used to calculate and interpret the overlap.^22^

## Results

### Review characteristics

This umbrella review included 16 reviews (2 scoping and 14 systematic reviews). At stage 1 screening, there was a 2% disagreement between reviewers and 0% at stage 2. Individual review characteristics are reported in online supplemental table 2.^58 23^,^36^ All reviews reported social prescribing research that included a link worker. Included reviews reported on 295 individual research articles, which were published between 1992 and 2023.^58 23^,^36^ Reviews included research conducted in Europe (n=16),^58 23^,^36^ North America (n=5),^24 26 29 35 36^ Australia (n=3),^23 32 35^ South America (n=1)^35^ and Asia (n=2).^26^

The total sample size was 106 556 participants reported in 15 reviews.^58 23^,^26 28^ One review did not report a total sample size.^34^ Eleven reviews reported on adults aged 18 years or older (range 18–90+years),^5 8 23 24 26 29 30 32 33 35^ one review focused on children (range 0–22 years),^36^ with four not reporting age data.^27 28 31 34^ In the six reviews that reported participants’ sex, there were more female participants than males (the percentage of female participants ranged from 36% to 100%).^24^,^2629 33 35^ The duration of social prescribing intervention was reported by six reviews and ranged from 1 to 84 months.^824^,^26 30 35^

Using the GROOVE tool to measure the overlap between primary studies reported by included reviews indicated a slight overlap in interpretation.^22^ However, the corrected covered area was 3.5%, which is below the threshold of <5% of slight overlap (lowest category). For GROOVE tool outputs, please see online supplemental table 3.

### Quality appraisal

**Figure 1:**
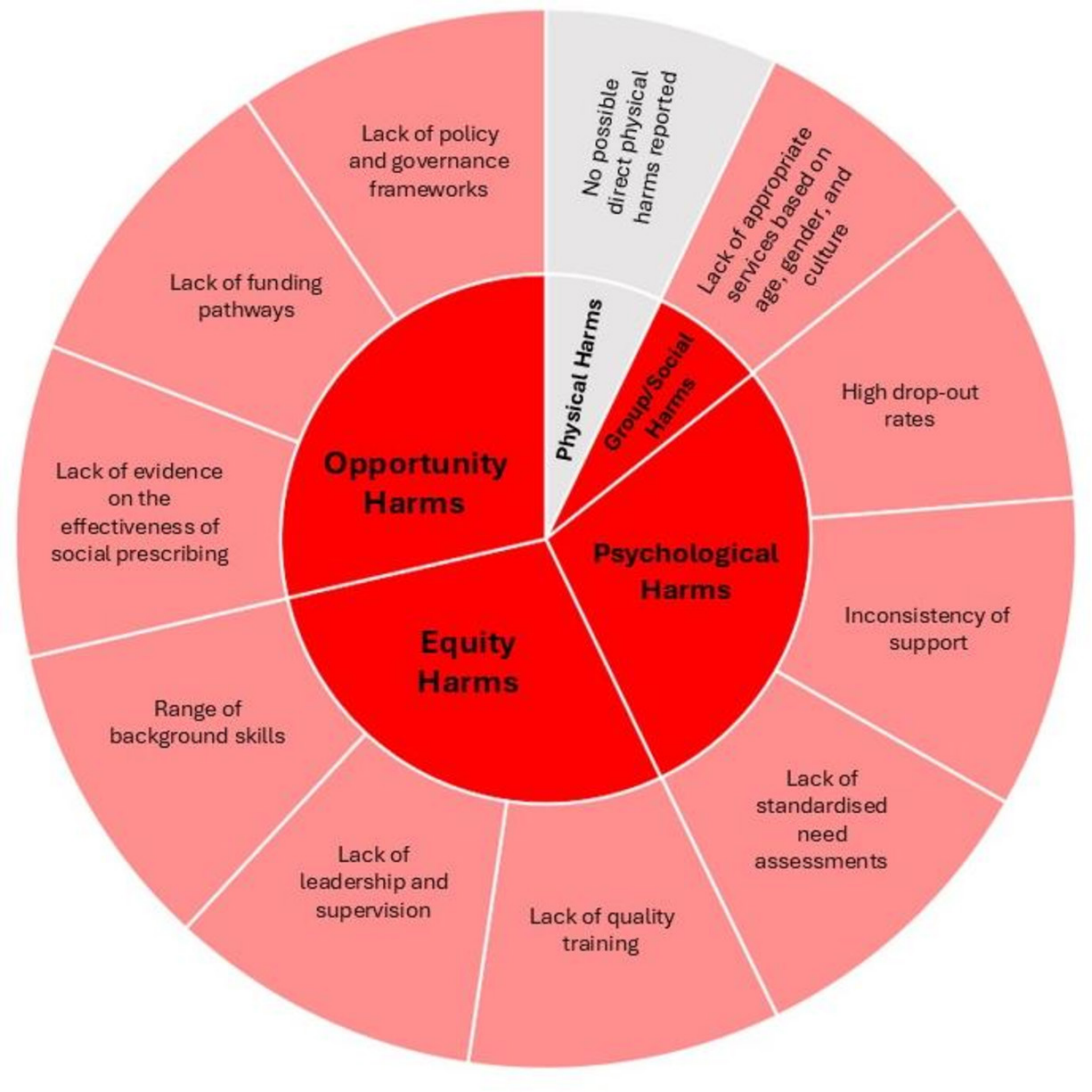
Summary of potential harms.

The average AMSTAR 2 score was 9.6 (SD=2.1, range 7–14). There were no conflicts between reviewers. One review scored the maximum score,^24^ with eight scoring below 10.^2325 28^,^30 34^ Two reviews used the components of population, intervention, comparator and outcome to define the inclusion criteria.^24 26^ Two reviews conducted an adequate investigation of publication bias and discussed the impact on the results of the review (although it should be acknowledged that this is not expected of scoping reviews).^23 24^ All reviews used at least two reviewers to perform review selection and data extraction.^58 11 23^,^32 34^ Full results of the quality appraisal can be viewed in online supplemental table 4 (figure 1).

### Physical harms

Direct physical harms were defined as ‘any adverse physical outcomes linked to prescribed activities (eg, injury, health deterioration)’. There were no data reported to suggest possible direct physical harms because of social prescribing in any of the reviews.

### Psychological harms

Referral to inappropriate services and the lack of continuity of care once referred to services can increase drop-out rates among social prescribing participants. High drop-out rates refer to the proportion of patients who stopped their involvement in an intervention (or in research) before its planned completion. In social prescribing practice, high drop-out rates can reduce the effectiveness of interventions, limit the achievement of intended health and well-being outcomes, and strain resources by requiring repeated engagement efforts. They may also act as a proxy for systemic barriers such as inadequate support, poor alignment with patient needs or accessibility challenges; ultimately, undermining trust in the approach and its sustainability.

Three reviews reported on the high drop-out observed in social prescribing, which was typically measured between referral and entry to social prescribing, entry to social prescribing and initial appointment or from initial appointment to onward support.^8 31 33^ Reviews reported a drop-out rate which ranged from 31% to 41% of patients enrolled on the intervention. The causes of the drop-out were under-reported within the reviews and there was no evidence to suggest this area had been explored further.^8 31 33^

However, one plausible contributing factor reported was the lack of continuity of care reported in three reviews.^8 23 30^ High workforce turnover led to inconsistent care for patients, with continuity and trust being recognised as central to the success of social prescribing. Frequent changes in link workers disrupted the therapeutic relationship, which was commonly built on rapport and personalised understanding of a patient’s social, emotional and practical needs. This inconsistency could result in fragmented care plans, repeated assessments and delays in connecting patients to appropriate community resources. Moreover, staff turnover could reduce institutional knowledge and weaken coordination between healthcare providers and community organisations; in turn, this is associated with diminished patient engagement and outcomes, as well as potential distal harm to patients due to inconsistency of support and how ‘participation in SP programs was disrupted by the high turnover of social prescribers*’*.^23^

Continuity was also affected by the lack of standardised needs assessment tools used by different services. The absence of standardised needs assessments in social prescribing resulted in inconsistent care because patient evaluations may vary significantly depending on individual link workers’ judgement, experience and available resources. Without a standardised approach, individual need could be overlooked or prioritised differently by different link workers (leading to inconsistent support). While standardised assessments have been regarded as essential for consistency and comparability, assessments must remain sufficiently flexible to accommodate individual circumstances, preferences and cultural contexts. A rigid approach risks reducing person-centredness and failing to address the psychology needs (leading to psychological harm). One review acknowledged the impact of COVID-19 on consistent delivery but also stated that ‘factors such as personnel changes … costly travel to referred activities, and referral delays resulted in a loss of engagement within social prescribing’.^30^

### Equity harms

Variation in how social prescribing services are delivered has been recognised as presenting possible equity harm. This stems from how services are designed (structural inequity in the design), but also includes the wide range of professional and non-professional backgrounds of social prescribers, a lack of or poor-quality training for new social prescribers and/or high variability in leadership and supervision (systemic inequity in provision). Within the included reviews, it was commonly reported that there was a wide range in the frequency of appointments that were offered.^25 28 30 35^ Five reviews reported the length of intervention offered ranged from 2 months^25^ to 2 years,^30^ with others having no maximum length of support.^26 30^ The total time available within appointments also varied, with some appointments lasting 10 min and others lasting up to 45 min.^28 35^

Social prescribers were identified to have varied professional backgrounds and with different levels of education.^35^ Consequently, this led to some social prescribers having skills in social work, mental health, community development or working with specific populations (patterned disparity based on equity of provision).^28^ Furthermore, seven reviews concluded that there was a lack of training for social prescribers, acknowledged as a risk of leading to an inequity of provision (systemic inequity).^823 26^,^30 35^ For example, differences in communication skills, knowledge of local resources and ability to build rapport could result in inconsistent patient experiences and inequitable access to appropriate services. Patients assigned to highly skilled link workers may receive comprehensive, tailored support, while others may experience fragmented care or limited options. This variability undermines the principle of equitable care and can distort the outcome of support, as improvements may reflect workforce capability rather than social prescribing intervention. This created plausible proximal harm to patients as they may receive support dependent on individual link worker competencies (inequity caused by institutional process and design of service).

Two reviews reported the wide variation in the length of training offered, reporting a range from a 2 hour workshop to a 16 week course.^26 28^ Not only did the length of training differ, but reviews that included social prescribers’ views concluded the training offered was inadequate (inequitable quality of provision).^2326^,^28^ Gaps in training for link workers were reported as referral processes,^23^ system navigation,^26^ engaging with the third sector^27^ and managing complex patients.^27^ Three reviews concluded that there was a lack of leadership and supervision offered to social prescribers,^23 28 30^ which, along with being a possible equity harm, could also be a potential opportunity harm. Although not directly concluded, the lack of supervision could also be linked to the high levels of burnout among social prescribers:

These intense levels of engagement and support come at a cost to link workers (social prescribers) who reported increasing tension and burnout from having to meet referral targets, arrange and deliver support, and help patients at crisis point.^30^

### Group or social harms

Numerous possible group or social harms were identified in the included reviews, particularly where social prescribing lacks provision of appropriate services based on age, gender and culture.^23 27 30 33 35 36^ Reviews concluded that social prescribing interventions did not offer programmes or support appropriate for the patient’s age which led to disengagement for many (see also psychological harms).^23 27 36^ It was noted that social prescribing research typically focused on adults aged 40 years and older, and services were established to support this age group.^25 28^ In addition to age-based exclusion, there was the potential for group or social harms due to a lack of appropriate cultural considerations within social prescribing. Reviews reported on how patients had faced difficulties with diet and healthy eating advice that was often based on Westernised norms, a lack of gender-specific exercise groups and language barriers within services.^23 27 30^

advice to participating in a social prescribing scheme may be understood differently by ethnic backgrounds. For example, some female patients from lower socioeconomic groups in a multi-ethnic urban setting reported that their partners generally do not allow them to leave the house or engage in mixed-sex group activities.^27^

Six reviews that included demographic information on sex included individual research reviews which included more female participants.^24^,^2629 33 35^ One review concluded that there was a noticeable lack of male participants in social prescribing research.^35^ No review provided evidence or suggestions as to the difference, and therefore this presents an additional possible group or social harm.

### Opportunity harms

The majority of possible harms relating to social prescribing were opportunity harms, which were also linked to equity and group or social harms. These opportunity harms often stem from the lack of evidence on the effectiveness of social prescribing either as a broad intervention or tailored to specific groups, as well as the existing funding pathways and the policy and governance frameworks that surround social prescribing interventions.

The lack of robust evidence to support the effectiveness of social prescribing was widely reported,^23^,^2631 34 36^ with reviews concluding that there was a lack of statistically significant improvements between premeasures and postmeasures.^24^,^2634 36^ Typically, health-focused interventions were evidence-driven to inform development, however there was a lack of improvements in patients post social prescribing. Three reviews found no difference in outcomes between routine care and social prescribing.^24 26 31^ Reviews investigating the effectiveness of supporting patients with mental health issues reported inconclusive findings.^24^,^2634^ Those reviews that focused on physical health reported that a greater proportion of individual reviews found no effect of social prescribing on physical health preintervention and postintervention.^24 26^ In addition to the overall immediate impact on patients, one review concluded that there was ‘no evidence about the effect social prescribing has on health and wellbeing outcomes beyond 6 months’.^31^ These suggested that there is an opportunity cost for patients who may be better served by a different or no intervention instead of an intervention that may not work.

These opportunity costs then also extended to resource allocation and funding,^5 23 24 27 31 33 36^ where, in contrast to the aims of social prescribing, two reviews concluded either healthcare costs went up postintervention or the cost of social prescribing did not offset the healthcare savings elsewhere in the system.^24 33^ Alternatively, four reviews concluded that there were insufficient funds available for social prescribing services, creating harms associated with a lack of sustainable provision of services (see psychological and equity harms).^23 24 27 33^ Failure to provide adequate funding for services led to a lack of resources within the community for onward referrals. The lack of funding and resources also directly impacted services as they could not grow capacity to expand the support available to patients of various backgrounds (see group/social harms).^23 27^ In addition, third sector organisations were affected as it was reported that they received little financial support despite increasing demand created by social prescribing on the services they provided.^27^

The lack of funding and resources did not just impact the patient, but also the perception others held of what social prescribing could support and the role social prescribers played. Three reviews reported on how social prescribing services often had to deal with inappropriate referrals from general practitioners (GPs), which they reported was due to a lack of knowledge about social prescribing.^5 23 31^ It was also reported that ‘GPs voiced concerns regarding the existence of unstructured (social prescribing) programs constrained by limited time and funding’.^23^ The reservations and inappropriate referrals from GPs impacted patient perceptions of social prescribing, setting unrealistic expectations.

Link workers across reviews reported insufficient investment and guidance in policy or governance of social prescribing.^23 27^ They felt that there was a lack of a clear and strategic approach to the integration with other forms of healthcare, leading to barriers to supporting patients effectively.^27^ The lack of policy and guidance equally impacted collaboration between social prescribers and the third sector, where there was ‘no real structure’ and that relied ‘on the goodwill of individuals’,^27^ which contributed to a lack of integrated care. One review concluded that:

strict bureaucracy and a ‘top-down’ approach within the healthcare system may hinder the appropriate involvement of different groups of stakeholders^27^

## Discussion

This umbrella review is the first review to categorise the potential harms associated with social prescribing and how the harms may arise. We have identified a range of potential harms though this is unlikely to be exhaustive, and we anticipate that other potential harms will exist within social prescribing but are yet unidentified due to the fledgling evidence base. The lack of research specifically examining potential harm in social prescribing initiatives, and typical challenges in identifying incidents that contribute to harm,^37^,^39^ means they are likely not identified or reported yet in social prescribing research. An example is the lack of any evidence identified on physical harms arising from social prescribing, which we consider a silence in the data rather than the possibility that no physical harms can arise during social prescribing activities. Limited evidence also means that we were unable to determine or comment on the incidence or prevalence of the harms identified in this umbrella review. It must also be acknowledged that social prescribing is a complex, multifaceted intervention that is fluid in delivery. The potential for harm is not static and instead will be dependent on the individual, link worker, need level, design, delivery and resource availability. This review provides the initial innovation in thinking to develop future thinking and how harm complexity can be reviewed. We highlight two clear outcomes:

1. The need for more robust design of social prescribing research, including collection of data on the incidence and prevalence of harms which are balanced against outcomes that measure success.
2. For designers and commissioners of social prescribing services to recognise and reflect on the potential for harm and to address these where practicable such as through the development of standardised training, consider equity in resource allocation decisions.

Of the identified harms, we found that opportunity harms were most reported in reviews. Many of the opportunity harms related to the lack of evidence on social prescribing, reflecting the relative infancy of social prescribing as an approach to holistic community healthcare. Consequently, definitions of social prescribing differ and there is significant heterogeneity in both the models of social prescribing and the services offered.^3^ Opportunity harms also arise due to methodological flaws in social prescribing research that are widely reported.^8^ Namely, attrition, theoretical underpinning and appropriate effectiveness assessment, impacting the ability to provide conclusive outcomes on cost-effectiveness.

Heterogeneity in social prescribing service design, delivery and inconsistency in what is offered and by whom presents possible equity and group/social harms. Health equity is increasingly recognised as influential in the fields of patient safety and personalised care as well as social prescribing.^16 40 41^ Alongside findings relating to variation in the training, support and leadership of link workers, this work has postulated that equity harms are unlikely to be identified in evaluations of single social prescribing services. There must also be consideration given to both the presentation and belief of the link worker role from other HCPs, which could be reflective of the individuals’ training. Instead, there is a need to examine equity harm on a larger scale, acknowledging possible influence of diversity in geographical location, populations, social determinants of health as well as the remit of social prescribing support offered. We also recognise that this same heterogeneity may allow services to develop and use workarounds to help facilitate access to services, thus providing possible enhancements in person-centred care and reductions in potential equity harms.^4042^,^44^ We believe this to be the case but suggest that without measuring this, the impact on equity is unknown, and it strengthens the case for future research on social prescribing interventions to give further consideration for potential equity harms.

Likewise, with the group harms where some services are tailored based on age, sex or ethnicity. While there is a wealth of evidence to suggest health declines as a result of ageing,^45^ there is a debate as to whether healthcare should be focused on improving the health of older age groups or focus on equity across the lifespan.^46^ Equally, it is important to ensure that services are tailored to the population’s needs and remove systemic barriers to inclusion due to ethnicity or sex. For social prescribing, there is a focus on community and connection to individuals with both similar and different backgrounds. Services should be inclusive of all individuals and look to celebrate diversity through coproduction and continued development. This has been suggested to improve both public engagement and diversity of participation.^47^,^49^

**Figure 2:**
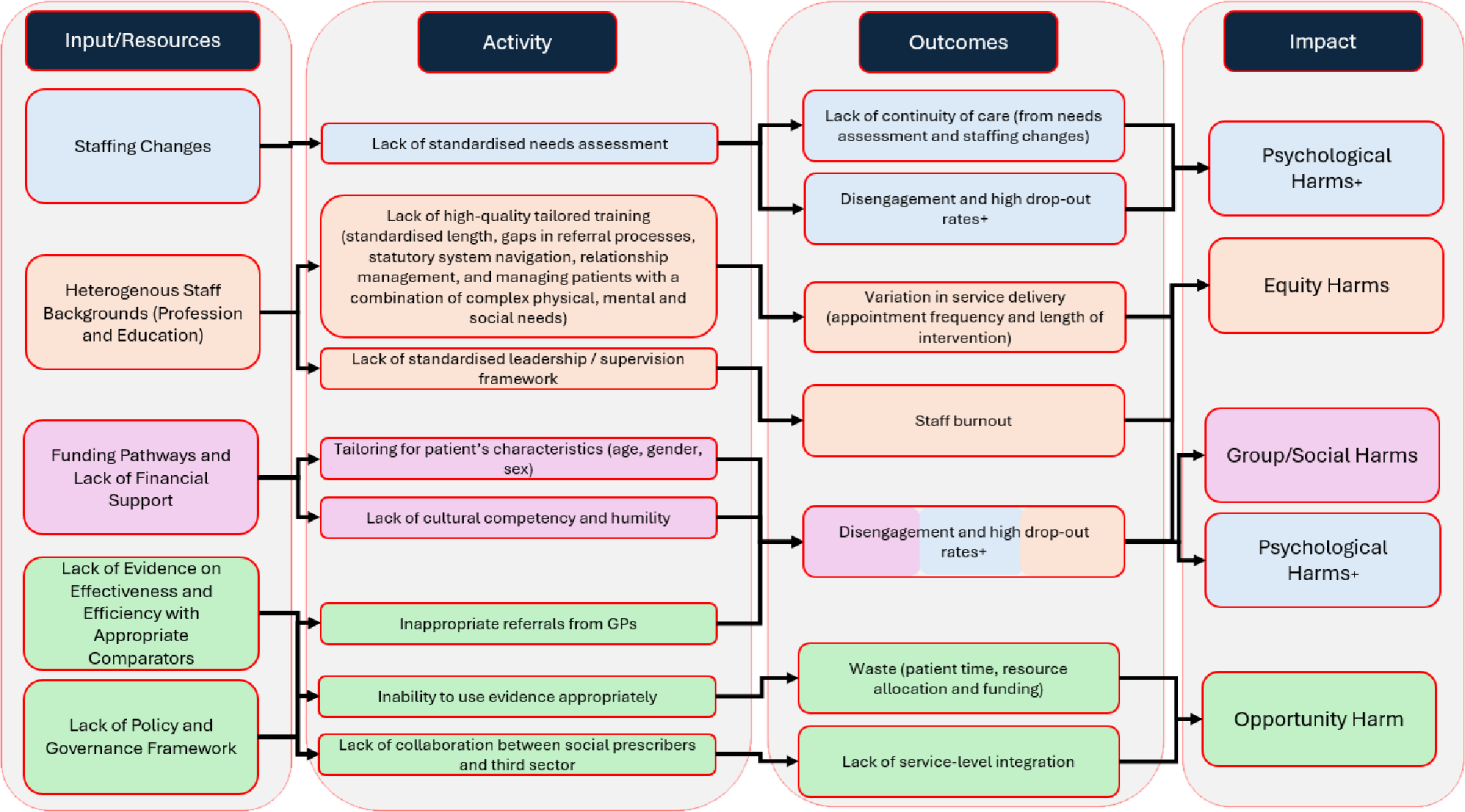
Dark logic model of social prescribing.

To illustrate the findings of this review and develop an understanding of how the potential harms may arise, a dark logic model of social prescribing has been developed (figure 2), offering a visual framework using an established dark logic framework of inputs and resources, activity, outcomes and impacts, the latter based on the typology of harms used in our coding.^50 51^ The dark logic of social prescribing highlights several ways in which social prescribing can lead to harm and gives indications for how the harms can be mitigated. For instance, the lack of high-quality evidence, which is widely reported^8^ and leads to inappropriate referrals and a lack of collaboration between services and service providers. This, in turn, can lead to resource waste and impacts on service-level integration of care, which leads to opportunity harms to the patient.

The dark logic model provides a starting point for people involved in the development, commissioning and delivery of social prescribing services to reflect on how harms may arise. In doing so, we anticipate that different stakeholders are likely to place emphasis on different harms. For instance, the social and group harms associated with social prescribing, linked to inconsistent funding pathways, are most likely relevant for policymakers, commissioners and service designers rather than those delivering a social prescribing service. There are also almost certainly gaps in the dark logic model, linked to the key finding of this review that there is limited evidence examining safety in social prescribing interventions.^14^,^16^ It is essential that future research tests the dark logic and further refines the model, such as including new harms and causal pathways where identified and removing existing harms or parts of their causal pathways where evidence indicates they do not exist. This would further help to evidence the incidence and prevalence of harms associated with social prescribing.

Future research should also consider the findings of this research in the context of prevention. Identification of evidence-informed potential harms to patients sets a clear need to address the issues raised and for future research to explore prevention strategies. This research highlights three main themes in particular: workforce, evidence base and resource management. We call for the development of standardised training, supervision frameworks and management for staff to ensure each link worker is supported in their role. Governance and policy are a key pillar of this but also should support the commissioning and evaluation of social prescribing. Consideration should be given to ensuring a long-term plan for resource management is sustainable and there is predefined governance to adhere to. Finally, the evidence base for social prescribing is growing, however, there needs to be a shift in how we evaluate effectiveness. Using single measures has proved inconclusive, and there needs to be more effort to creatively and robustly measure change (eg, a visual self-assessment tool where patients rate their health across the three dimensions of health, social, physical and mental).

### Limitations

There is a lack of a globally recognised definition of social prescribing, and often no direct translation in many languages. While there has been consensus work completed to develop a universally accepted definition,^3^ this was only published in 2023 and does not have widespread adoption. This limits the evidence included in this review to some degree, where alternative definitions could have been missed or excluded.

Identification of harms is notoriously difficult, often requiring multiple methods that each have their limitations.^13 50^ In this review, we attempted to categorise harms by conducting an umbrella review on social prescribing and thus relied on the accuracy and quality of the reviews to capture evidence on harms. While we assessed the quality of all included reviews, it is possible that the reviews themselves did not attempt to identify potential harms and thus may bias the results. This review did not exclude reviews based on synthesis of outcomes of harms as it was known to the authors this has never been attempted in current social prescribing literature. While there was data to suggest harms, it is not an exhaustive review but provides a justification of why future research is needed. This review should be considered the first step in identifying harms associated with social prescribing and provide the basis for future primary research.

It must also be acknowledged the overlap between different types of harm within the Typology of Harms framework. While we applied clearly defined definitions of harm, the likelihood that harms exist in isolation is reductionist. We acknowledge that the evidence available has been coded against certain definitions but there is future data required to establish the connectivity within the framework, and therefore a limitation of this review.

### Conclusions

Social prescribing, as with any innovation in the organisation and delivery of care, has the potential to cause harm. This is the first review to categorise the potential harms associated with social prescribing. We have identified a range of potential harms, categorised as either a psychological, group/social, equity or opportunity harm. However, it is unlikely to be exhaustive, and we anticipate that other potential harms will exist within social prescribing, but future research is needed. We provide two clear outcomes: (1) the need for more robust design of social prescribing research, including collection of data on the incidence and prevalence of harms, and (2) recognition of the potential for harm from social prescribing and to address these where practicable.

## Supplementary material

10.1136/bmjopen-2025-108998 online supplemental file 1

10.1136/bmjopen-2025-108998 online supplemental file 2

10.1136/bmjopen-2025-108998 online supplemental file 3

10.1136/bmjopen-2025-108998 online supplemental file 4

## Data Availability

Anonymised data used and/or analysed during the current review are available upon reasonable request from the corresponding author.
