# Supplementary material for: Potential harms of social prescribing: a global umbrella review and dark logic model
Source: BMJ Open. 2026 May 4;16(5):e108998. doi: 10.1136/bmjopen-2025-108998 (PMC13141156; doi:10.1136/bmjopen-2025-108998)
Supplement: online supplemental file 2 [file bmjopen-16-5-s002.docx]

Supplementary Material 2

Summary Table of Included Reviews

| Ref No. | Author | Research Design | Date range | n | Age Range | Females % | Males % | Countries Included | Contract Duration | No. refs. |
| --- | --- | --- | --- | --- | --- | --- | --- | --- | --- | --- |
| 23 | Yadav et al,. | Rapid | 2017-2023 | 10379 | Range 40-74 | _ | _ | UK, Australia | _ | 15 |
| 24 | Kiely et al | Systematic | 1992-2022 | 6500 | Range 29-71 | 59-75 | 25-41 | UK, USA | Range 1m to 24m | 9 |
| 5 | Cooper et al,. | Systematic | 2008-2022 | 5036 | Range 43-77 | _ | _ | UK | Range 1.5m to 9m | 17 |
| 25 | Costa et al,. | Systematic | 2000-2019 | 4603 | Range 49- 80 | 46-82 | 18-54 | UK | Range 9m to 84m | 13 |
| 26 | Teggart et al,. | Systematic | 2009-2020 | 10743 | Range 49-82 | 54- 77 | 24-46 | China, UK, USA, Sweden, Netherlands, Greece, Croatia, Spain, Canada | 2m to 30m | 21 |
| 27 | Ebrahimoghli et al,. | Systematic | 2004-2020 | 578 | _ | _ | _ | UK, Norway, Netherlands, Spain | _ | 22 |
| 28 | Sandhu et al,. | Scoping | 2004-2021 | 13829 | _ | _ | _ | UK | _ | 32 |
| 29 | Grover et al,. | Systematic | 2014-2021 | 197 | Range 29-91 | 59 | 41 | Canada, England, Ireland and Netherlands | _ | 8 |
| 30 | Linceviciute et al,. | Systematic | 2017-2023 | 1131 | Range 26-90+ | _ | _ | UK, Ireland | Range 3-24m | 18 |
| 8 | Cooper et al,. | Systematic | 2012-2021 | 220 | Range 47-77 | _ | _ | England, Wales, Scotland | _ | 6 |
| 31 | Bickerdike et al,. | Systematic | 2000-2016 | 900 | _ | _ | _ | UK | _ | 15 |
| 32 | Napierala et al,. | Systematic | 2014-2021 | 41627 | Range 18-95 | _ | _ | UK, Australia |  | 53 |
| 33 | Percival et al,. | Systematic | 2014-2021 | 546 | Range 64-94 | 36-100 | 0-64 | Northern Ireland, England, Wales, South Korea | _ | 7 |
| 34 | Pescheny et al,. | Systematic | 2000-2017 | - | _ | _ | _ | UK | _ | 16 |
| 35 | O’Grady et al,. | Scoping | 2001-2021 | 10,104 | Mean 58.1 (SD9.9) | 47 | 43 | UK, USA, Canada, Netherlands, Spain, Denmark, Australia, Brazil | 0-6m | 35 |
| 36 | Gordon et al,. | Systematic | 2004-2018 | 163 | Range 0-22 | _ | _ | UK, USA, France | _ | 8 |
