# Supplementary material for: Potential harms of social prescribing: a global umbrella review and dark logic model
Source: BMJ Open. 2026 May 4;16(5):e108998. doi: 10.1136/bmjopen-2025-108998 (PMC13141156; doi:10.1136/bmjopen-2025-108998)
Supplement: online supplemental file 3 [file bmjopen-16-5-s003.docx]

Supplementary Materials 3

|  |  |  |
| --- | --- | --- |
| Number of columns (number of reviews) | c | 16 |
| Number of rows (number of index publications) | r | 197 |
| Number of included primary studies (including double counting) | N | 300 |
| Covered area | N/(rc) | 9.52% |
| Corrected covered area | (N-r)/(rc-r) | 3.49% |
| Interpretation of overlap | **Slight overlap** | |
| Structural Zeros | X | 0 |
| Corrected covered area  (adjusting by structural zeros) | (N-r)/(rc-r-X) | 3.49% |

GROOVE Tool Outputs

| N° of non-overlapped primary studies | In 1 SR | 117 |
| --- | --- | --- |
| Number of overlapped primary studies | In 2 SRs | 18 |
|  | In 3 SRs | 13 |
|  | In 4 SRs | 2 |
|  | In 5 SRs | 3 |
|  | In 6 SRs | 4 |
|  | In 7 SRs | 1 |
|  | In 8 SRs | 1 |
|  | In 9 SRs | 1 |
|  | In 10 SRs | 0 |
|  | In 11 SRs | 0 |
|  | In 12 SRs | 0 |
|  | In 13 SRs | 0 |
|  | In 14 SRs | 0 |
|  | In 15 or more SRs | 0 |

|  | Napierela 2022 | O'Grady 2024 | Sandhu 2022 | Ebrahimoghli 2025 | Teggart 2023 | Cooper 2022 | Bickerdike 2017 | Yadav 2024 | Linceviciute 2023 | Pescheny 2020 | Costa 2021 | Kiely 2022 | Grover 2023 | Gordon 2023 | Percival 2022 |
| --- | --- | --- | --- | --- | --- | --- | --- | --- | --- | --- | --- | --- | --- | --- | --- |
| O'Grady 2024 | 1.1% |  |  |  |  |  |  |  |  |  |  |  |  |  |  |
| Sandhu 2022 | 10.4% | 3.1% |  |  |  |  |  |  |  |  |  |  |  |  |  |
| Ebrahimoghli 2025 | 2.7% | 3.6% | 8.2% |  |  |  |  |  |  |  |  |  |  |  |  |
| Teggart 2023 | 8.2% | 3.4% | 7.7% | 0.0% |  |  |  |  |  |  |  |  |  |  |  |
| Cooper 2022 | 7.6% | 0.0% | 4.3% | 2.6% | 7.7% |  |  |  |  |  |  |  |  |  |  |
| Bickerdike 2017 | 14.8% | 0.0% | 9.3% | 2.7% | 0.0% | 0.0% |  |  |  |  |  |  |  |  |  |
| Yadav 2024 | 1.5% | 2.0% | 15.0% | 12.1% | 2.6% | 3.2% | 0.0% |  |  |  |  |  |  |  |  |
| Linceviciute 2023 | 5.9% | 1.9% | 16.7% | 8.1% | 4.9% | 0.0% | 0.0% | 26.9% |  |  |  |  |  |  |  |
| Pescheny 2020 | 18.6% | 2.0% | 11.9% | 5.6% | 5.1% | 3.1% | 39.1% | 6.9% | 3.0% |  |  |  |  |  |  |
| Costa 2021 | 15.5% | 0.0% | 18.9% | 2.9% | 18.8% | 25.0% | 7.4% | 3.7% | 6.9% | 20.8% |  |  |  |  |  |
| Kiely 2022 | 4.9% | 0.0% | 7.9% | 3.2% | 6.1% | 3.8% | 4.0% | 4.2% | 3.7% | 8.3% | 15.0% |  |  |  |  |
| Grover 2023 | 3.3% | 2.4% | 8.3% | 7.1% | 3.1% | 4.2% | 0.0% | 15.0% | 13.0% | 9.1% | 10.5% | 0.0% |  |  |  |
| Gordon 2023 | 0.0% | 0.0% | 0.0% | 0.0% | 0.0% | 0.0% | 0.0% | 0.0% | 0.0% | 0.0% | 0.0% | 0.0% | 0.0% |  |  |
| Percival 2022 | 5.3% | 0.0% | 8.8% | 0.0% | 3.3% | 4.5% | 0.0% | 0.0% | 4.3% | 10.0% | 11.8% | 0.0% | 7.7% | 0.0% |  |
| Cooper 2023 | 1.7% | 2.5% | 8.8% | 7.7% | 3.3% | 4.5% | 0.0% | 16.7% | 14.3% | 4.8% | 5.6% | 0.0% | 27.3% | 0.0% | 0.0% |
