# Supplementary material for: Potential harms of social prescribing: a global umbrella review and dark logic model
Source: BMJ Open. 2026 May 4;16(5):e108998. doi: 10.1136/bmjopen-2025-108998 (PMC13141156; doi:10.1136/bmjopen-2025-108998)
Supplement: online supplemental file 4 [file bmjopen-16-5-s004.docx]

Supplementary Materials 4

Quality Appraisal Table

|  |  | 1 | 2 | 3 | 4 | 5 | 6 | 7 | 8 | 9 | 10 | 11 | 12 | 13 | 14 | 15 | 16 |
| --- | --- | --- | --- | --- | --- | --- | --- | --- | --- | --- | --- | --- | --- | --- | --- | --- | --- |
| Yadav UN, Paudel G, Ghimire S, et al. A rapid review of opportunities and challenges in the implementation of social prescription interventions for addressing the unmet needs of individuals living with long-term chronic conditions. BMC public health 2024;24(1):306. |  | N | N | N | Y | Y | Y | Y | Y | N | Y | N/A | N/A | N | N | Y | Y |
| Kiely B, Croke A, O'Shea M, et al. Effect of social prescribing link workers on health outcomes and costs for adults in primary care and community settings: a systematic review. BMJ open 2022;12(10):e062951. |  | Y | Y | Y | Y | Y | Y | Y | Y | Y | Y | N/A | N/A | Y | Y | Y | Y |
| Cooper M, Avery L, Scott J, et al. Effectiveness and active ingredients of social prescribing interventions targeting mental health: a systematic review. BMJ Open 2022;12(7):e060214. |  | N | Y | Y | Y | Y | Y | Y | Y | Y | N | N/A | N/A | Y | Y | N | Y |
| Costa A, Sousa CJ, Seabra PRC, et al. Effectiveness of social prescribing programs in the primary health-care context: a systematic literature review. Sustainability 2021;13(5):2731. |  | N | Y | Y | Y | Y | Y | N | N | Y | Y | N/A | N/A | Y | Y | N | N |
| Teggart K, Neil-Sztramko SE, Nadarajah A, et al. Effectiveness of system navigation programs linking primary care with community-based health and social services: a systematic review. BMC Health Services Research 2023;23(1):450. |  | Y | Y | Y | Y | Y | Y | Y | Y | Y | Y | N/A | N/A | Y | Y | N | Y |
| Ebrahimoghli R, Pezeshki M, Farajzadeh P, et al. Factors influencing social prescribing initiatives: a systematic review of qualitative evidence. Perspectives in Public Health 2023:17579139231184809. |  | N | Y | Y | N | Y | Y | Y | Y | Y | Y | N/A | N/A | Y | Y | N | Y |
| Sandhu S, Lian T, Drake C, et al. Intervention components of link worker social prescribing programmes: a scoping review. Health & social care in the community 2022;30(6):e3761-e74. |  | N | N | Y | Y | Y | Y | Y | Y | N^+^ | N | N/A | N/A | N | Y | N | Y |
| Grover S, Sandhu P, Nijjar GS, et al. Older adults and social prescribing experience, outcomes, and processes: a meta-aggregation systematic review. Public Health 2023;218:197-207. |  | N | N | N | Y | Y | Y | Y | N | N | Y | N/A | N/A | N | Y | N | Y |
| Linceviciute S, Ambrosio L, Baldwin DS, et al. Role of Social Prescribing Link Workers in Supporting Adults with Physical and Mental Health Long‐Term Conditions: Integrative Review. Health & Social Care in the Community 2023;2023(1):7191247. |  | N | N | Y | Y | Y | Y | Y | Y | N | N | N/A | N/A | N | Y | N | Y |
| Cooper M, Flynn D, Avery L, et al. Service user perspectives on social prescribing services for mental health in the UK: a systematic review. Perspectives in Public Health 2023;143(3):135-44. |  | N | Y | N | Y | Y | Y | Y | Y | Y | N | N/A | N/A | Y | Y | N | Y |
| Bickerdike L, Booth A, Wilson PM, et al. Social prescribing: less rhetoric and more reality. A systematic review of the evidence. BMJ open 2017;7(4):e013384. |  | N | Y | Y | Y | Y | Y | Y | Y | Y | N | N/A | N/A | Y | Y | N | Y |
| Napierala H, Krüger K, Kuschick D, et al. Social prescribing: systematic review of the effectiveness of psychosocial community referral interventions in primary care. International journal of integrated care 2022;22(3):11. |  | N | Y | Y | Y | Y | Y | Y | Y | Y | N | N/A | N/A | Y | Y | N | Y |
| Percival A, Newton C, Mulligan K, et al. Systematic review of social prescribing and older adults: where to from here? Family medicine and community health 2022;10(Suppl 1):e001829. |  | N | N | Y | Y | Y | Y | Y | Y | Y | Y | N/A | N/A | Y | Y | N | Y |
| Pescheny JV, Randhawa G, Pappas Y. The impact of social prescribing services on service users: a systematic review of the evidence. European journal of public health 2020;30(4):664-73. |  | N | N | Y | Y | Y | Y | Y | Y | N | N | N/A | N/A | N | N | N | Y |
| O’Grady M, Connolly D, Kennedy M, et al. The role of intermediaries in connecting community-dwelling adults to local physical activity and exercise: a scoping review. International Journal of Integrated Care 2024;24(2):12. |  | N | Y | N | Y | Y | Y | Y | Y | N^+^ | N | N/A | N/A | N | Y | N | N |
| Gordon K, Gordon L, Basu AP. Social prescribing for children and young people with neurodisability and their families initiated in a hospital setting: a systematic review. BMJ open 2023;13(12):e078097. |  | N | Y | Y | Y | Y | Y | Y | Y | N | N | N/A | N/A | N | N | N | Y |
| Yadav UN, Paudel G, Ghimire S, et al. A rapid review of opportunities and challenges in the implementation of social prescription interventions for addressing the unmet needs of individuals living with long-term chronic conditions. BMC public health 2024;24(1):306. |  | N | N | N | Y | Y | Y | Y | Y | N | Y | N/A | N/A | N | N | Y | Y |

+ not expected practice of scoping reviews.

AMSTAR 2: Questions.

1. Did the research questions and inclusion criteria for the review include the components of PICO?
2. Did the report of the review contain an explicit statement that the review methods were established prior to the conduct of the review and did the report justify any significant deviations from the protocol?
3. Did the review authors explain their selection of the study designs for inclusion in the review?
4. Did the review authors use a comprehensive literature search strategy?
5. Did the review authors perform study selection in duplicate?
6. Did the review authors perform data extraction in duplicate?
7. Did the review authors provide a list of excluded studies and justify the exclusions?
8. Did the review authors describe the included studies in adequate detail?
9. Did the review authors use a satisfactory technique for assessing the risk of bias (RoB) in individual studies that were included in the review?
10. Did the review authors report on the sources of funding for the studies included in the review?
11. If meta-analysis was performed did the review authors use appropriate methods for statistical combination of results?
12. If meta-analysis was performed, did the review authors assess the potential impact of RoB in individual studies on the results of the meta-analysis or other evidence synthesis?
13. Did the review authors account for RoB in individual studies when interpreting/ discussing the results of the review?
14. Did the review authors provide a satisfactory explanation for, and discussion of, any heterogeneity observed in the results of the review?
15. If they performed quantitative synthesis did the review authors carry out an adequate investigation of publication bias (small study bias) and discuss its likely impact on the results of the review?
16. Did the review authors report any potential sources of conflict of interest, including any funding they received for conducting the review?
