# Supplementary material for: Potential harms of social prescribing: a global umbrella review and dark logic model
Source: BMJ Open. 2026 May 4;16(5):e108998. doi: 10.1136/bmjopen-2025-108998 (PMC13141156; doi:10.1136/bmjopen-2025-108998)
Supplement: Supplementary file 5 [file bmjopen-2025-108998.reviewer_comments.pdf]

PEER REVIEW HISTORY

BMJ Open publishes all reviews undertaken for accepted manuscripts. Reviewers are asked to complete a checklist review form and are provided with free text boxes to elaborate on their assessment. These free text comments are reproduced below.

ARTICLE DETAILS

Title (Provisional)

The Potential Harms of Social Prescribing. A Global Umbrella Review and Dark Logic Model.

Authors

Cooper, Matthew; Okeowo, Daniel; Bennett, Liesel; Aslam, Eman; Yahya, Faiza; Lawson, Lauren; Robinson-Barella, Anna; Nazar, Hamde; Scott, Jason

---

VERSION 1 - REVIEW

**Reviewer**	**1**
**Name**	**Tierney, Stephanie**
**Affiliation**	**University of Oxford, Nuffield Department of Primary Care**
**Health Sciences**	
**Date**	**26-Aug-2025**
**COI**	**None**

This review of reviews covers an interesting topic – potential harms of social prescribing. My main concern is that several reviews seem to be missing, including:

- Tierney et al. (2020) https://bmcmedicine.biomedcentral.com/articles/10.1186/s12916-020-1510-7
- Husk et al. (2020) https://pubmed.ncbi.nlm.nih.gov/31502314/
- Handayani et al. (2024) https://pubmed.ncbi.nlm.nih.gov/39266243/
- Napierala et al. (2022) https://pubmed.ncbi.nlm.nih.gov/36060831/

There are several other areas that I think the authors need to consider:

- Abstract
  - Needs to be clear that psychological and equity harms are different
- Introduction
  - Was it the UK NHS or NHS in England – the reference seems to be NHS in England?
  - Mentions dark logic – the authors need to explain what this is
- Methods

    - How was coding of data undertaken? By hand or some computer system?
- Results
    - Under psychological harms – is a needs assessment right for everyone? Could bring up issues that people don't want to discuss with a link worker and, therefore, to ask might cause harm. Link workers have talked about their dislike of using standardised measures because they are seen as impersonal and break the rapport they are trying to develop with patients. Any standardised needs assessment would need to be sensitive to this.
    - I was not completely clear what opportunity constituted when written about in the results from line 260 onwards – perhaps this could be made clearer.
    - There might be something about training and backgrounds of link worker and how this shapes how other professionals see this role. An individual link worker might come to be reflective, of all link workers to health professionals they interact with (for good or bad). But what they do as an individual may be shaped by their background and training (or lack thereof) as well as resource constraints and system level factor.
    - I was surprised there was nothing about dependency on link workers – patients becoming dependent on them?
    - Or nothing about burnout of link workers? Or of safeguarding risks and link workers taking on cases they are not trained to support?
- Discussion
    - There seemed to be quite a lot of overlap between different types of harms – perhaps this could be alluded to in the discussion?
    - The authors used the Typology of Harms to help categorise data – it would be good to reflect on the strengths and limitations of having done so.
    - The authors suggest a need for more creative ways to assess outcomes at the end of the discussion (line 397) – it would be good to have some thoughts on this and suggestions of what this might look like?
    - In terms of limitations – the authors might reflect on this being a review of reviews so some relevant primary papers might not have been picked up a) because they were not included in the reviews that were included, or b) they were included in reviews but the sections on harms in these primary papers were not extracted into the reviews.
- Tables
    - Table 2 – I could not work out what the system was – usually articles in a table would be in alphabetical or chronological order – but I couldn't work out what system was used here.
    - I feel there needed to be a table of included reviews – a table of characteristics of the reviews.

**Reviewer**	**2**
**Name**	**Jonsson, Frida**
**Affiliation**	**Umeå Universitet Medicinska fakulteten, Department of Social Work**
**Date**	**13-Sep-2025**
**COI**	**None**

Thank you for giving me the opportunity to review this article titled "The Potential Harms of Social Prescribing. A Global Umbrella Review and Dark Logic Model." While the study is interesting and has the potential to fill an important knowledge gap, there are several aspects that make the manuscript fall short of that potential. Below are some comments for the authors to consider in light of this.

Rather than presenting evidence of actual harms as reported in the included reviews, the authors generally provide evidence on different factors that may contribute to different harms outlined in the typology of harms framework. For example, instead of providing evidence on widening of inequity (although vague what that means since the authors do not elaborate on the framework), they identify different conditions that could lead to "inequity" (unclear of what). While this would be fine and interesting, it requires more conceptual and theoretical work by the authors to properly reason and explain how and why the different conditions may result in different harms. One solution to this could be to structure the results around the dark logic model rather than thematically around the harms.

However, regardless of the focus chosen, the potential harms need to be more properly defined conceptually (e.g., what does psychological and equity harms mean?) and detailed empirically (i.e., what psychological or equity harms were identified in the literature, not just conditions that contribute broadly to "psychological harms").

Overall, I have a hard time following the results since I do not have a clear understanding of the typology of harms framework after reading the manuscript. The authors should include a conceptual framework section where the typology of harms is presented and the five categories of adverse effects of interventions are properly defined and described. In addition, the equity and social/group harms seems conceptually very similar, so the authors should clarify how they differ. The text would also benefit from including some information on contexts/topics in which this framework has been previously used.

1. Please define and describe what a dark logic model is when first mentioned. The different components of logic models should also be defined and the process of developing the dark logic model explained.

2. The umbrella review design should be described, and the authors should provide a rationale for conducting this specific type of review.

3. The authors state that “no possible direct physical harms were reported in any of the reviews”. Please explain what this means.

4. The authors should provide some evidence for the statement “High drop-out rates caused by referral to inappropriate services and the lack of continuity of care once referred to services increase the risk of causing proximal psychological harm to patients”. Alternatively, they should explain in more detail what this means, so the reader can understand the reasoning. What does high drop-out rates mean? Referral to inappropriate services and the lack of continuity of care? Proximal psychological harms?

5. The paragraph on potential psychological harm is vague and somewhat speculative, providing neither tangible evidence nor a proper reasoning on the links between drop-out, lack of care continuity, lack of standardised needs assessment, and staff turnover to psychological harm (which is not properly explained). Hence, after reading this manuscript, I am not convinced about the potential psychological harms of social prescribing.

6. The statement, “this created skill gaps in service provision, creating plausible proximal harm to patients”, needs to be elaborated. Creating plausible proximal harm to patients, how? Why?

Similarly, the authors state that “seven reviews concluded there was a lack of training for social prescribers. [sic] leading to an inequity of provision” – how does lack of training lead to inequity of provision?

7. Revise the sentence: “the framework was used to provide structure to the data extraction and provide guidance to data extraction”.

**Reviewer**	**3**
**Name**	**Marshall, Jessica**
**Affiliation**	**University of East Anglia, Health Sciences**
**Date**	**06-Oct-2025**
**COI**	**None**

Comments to Author

Thank you for the opportunity to review this manuscript, “The Potential Harms of Social Prescribing: A Global Umbrella Review.” I am delighted to see a comprehensive review in this important and emerging field. This umbrella review addresses a critical knowledge gap, is well written, and effectively integrates dark logic modelling with the Typology of Harms Framework to enhance the interpretation of findings. The topic is highly relevant, and the paper’s insights have the potential to significantly influence future research and inform the design of social prescribing (SP) interventions.

However, there are several areas that could be strengthened, particularly regarding the clarity of the methods, the presentation of results, and discrepancies between the protocol and this manuscript, which to my knowledge have not been amended in PROSPERO. I have outlined specific suggestions below, which I hope the authors find constructive.

Abstract:

- The abstract is clear and well structured; however, it would benefit from mentioning the use of dark logic modelling within the methods.

- In the results section, please highlight that no physical harms were identified, this is an important and notable finding.

- Rather than defining the five harm categories in the abstract, focus on illustrative examples of actual findings within each (e.g. group social harms such as age-based exclusion, language barriers, gender-specific groups, overly Westernised approaches).

Introduction:

- The introduction provides a strong overview and clearly defines SP. However, please include an early definition of “link worker” (thank you for noting that the role is known by various titles) . It is important to be explicit about how this term is defined for the purposes of the review as primary care vs third sector definitions vary.

- Some cited studies apply to specific patient groups only (e.g., the Cooper reference to mental health (line 64-66), the Skinner reference to Type 2 diabetes (line 67-70)). Please clarify this to avoid overgeneralisation.

- Consider expanding on how dark logic modelling is applied within the review. It is mentioned briefly in the introduction and conclusion, but it may warrant a dedicated paragraph in the methods describing what it is, how it was used, why it was chosen, and how it has been used in integration with the Typology of Harms framework. This is a strength of the review and deserves to be showcased.

Methods:

- I note the protocol is published in PROSPERO which I have looked at.

- Did the authors consider including the preferred reporting items for overviews of reviews (PRIOR) statement to understand the trustworthiness and applicability of their findings. The PRIOR statement is endorsed by latest umbrella review guidelines (Fernandez et al. 2025 https://doi.org/10.1093/eurjcn/zvaf012)

- The inclusion criteria could be strengthened by outlining the PICO framework here, rather than only in the quality appraisal section.

- The justification for the 2010 start date (line 114) should be amended, as SP was referenced earlier (e.g., Friedli & Watson, 2004; South et al., 2008, doi:10.1017/S146342360800087X).

- Consider including an example search string, as terms are not currently outlined in the manuscript or PROSPERO record.

- The data extraction criteria described in the manuscript appear to differ from those in the PROSPERO protocol. Please clarify whether all variables listed in the protocol were extracted. If not, explain and justify any deviations.

- The explanation of the AMSTAR 2 tool is clear.

- The data synthesis section should indicate whether the planned categorisation (patient safety vs. healthcare system impact) was completed. If so, please report the findings accordingly.

- During synthesis, clarify whether quantitative and qualitative data were analysed separately or integrated, and justify the approach.

- Please explain how study overlap was managed - for example, whether a tool such as GROOVE (10.1002/jrsm.1557) was used.

Results:

- A flowchart of the literature search and study selection would be helpful (https://bmjmedicine.bmj.com/content/1/1/e000071)

- A summary table of study characteristics should be included in the main text or supplementary materials. It is mentioned as Supplementary Material 1, but not visible in the version provided. Also, on this point, regarding data extraction umbrella reviews "researchers should extract the number of eligible studies, the total sample size and (for binary outcomes) the number of events, the rationale for not performing a meta-analysis, and the descriptive conclusions" (https://bmjmedicine.bmj.com/content/1/1/e000071) – I'm not sure whether this was done or not because I cannot see the extraction table.

- The results are good and well-mapped to the Typology of Harm framework. However, it may be helpful for the reader if the results could be separated into patient safety, impact on those who deliver SP (link workers), and the impact on current health systems. This will help demonstrate which harms affect the specific population groups that are included in this umbrella review (patients, link workers)

Discussion

- The discussion could be strengthened by linking findings more explicitly to the complexity of SP as a multifaceted intervention.

- The protocol indicates that subgroup analyses (e.g., intervention types, pre–post outcomes) were planned. If these data were unavailable, please reflect on why this may be the case.

- Regarding the limitations, I would argue that the 2023 Muhl definition is established and adopted widely in the literature. The definition was coined through a rigorous Delphi study, it would be hard to justify the use of any other definition currently.

- Consider adding study overlap as a limitation

General

- Good logical layout and clear written English used. Referencing is good.

**Reviewer**	**4**
**Name**	**Schoenmakers, Birgitte**
**Affiliation**	**KU Leuven, Public Health and Primary Care**
**Date**	**28-Oct-2025**
**COI**	**None**

Thank you for sharing this well-developed scoping review. I read it with great interest and even found myself exploring the concept of “dark logic models” further.

However, several issues arose during my reading and review:

• The term dark logic is introduced but not clearly distinguished from general risk or harm frameworks. It remains unclear how this model offers additional explanatory value beyond the existing Typology of Harms Framework.

• The model seems largely deductive and speculative. I found limited evidence in the included reviews to support the construction of causal chains of harm.

• Therefore, I recommend clarifying the theoretical contribution of the dark logic model: is it intended as a novel framework, an adaptation of existing models, or a visual summary?

Regarding the search strategy and the outcomes included, I offer the following observations:

• The absence of reported physical harms is interpreted as a “silence in the data,” which feels speculative. It would be more robust to acknowledge the limitations of the included reviews in identifying such harms.

• Psychological and opportunity harms are well described, but often lack quantitative data on prevalence or severity.

• The narrative synthesis is rich, but could be strengthened by more systematic coding or tabulation.

• Equity and group/social harms are discussed in terms of variability in service provision, yet the connection to actual patient outcomes appears weak. Many harms are inferred rather than directly reported.

To enhance methodological transparency, I suggest clarifying how harms were extracted, coded, and synthesized. Additionally, consider including a supplementary table that maps each harm category to specific examples from the included reviews, with appropriate citations and contextual information.

VERSION 1 - AUTHOR RESPONSE

Reviewer 1	
This review of reviews covers an interesting topic – potential harms of social<br>prescribing. My main concern is that several reviews seem to be missing, including:<br>• Tierney et al. (2020) https://bmcmedicine.biomedcentral.com/articles/10.1186/s12916-020-1510-7<br>• Husk et al. (2020) https://pubmed.ncbi.nlm.nih.gov/31502314/<br>• Handayani et al. (2024) https://pubmed.ncbi.nlm.nih.gov/39266243/<br>• Napierala et al. (2022) https://pubmed.ncbi.nlm.nih.gov/36060831/	Thank you for highlighting these reviews. While Tierney, Husk, and Napierala were picked up in the search, they were excluded as they included non-peer reviewed data. For clarity we have updated the inclusion criteria as follows: *which synthesised peer-reviewed primary data collected from any person in receipt, involved in delivery, or the commissioning of social prescribing interventions (defined previously).*<br><br>In relation to Handayani, this review was published after the search was conducted.
Abstract<br>o Needs to be clear that psychological and equity harms are different	Thank you this has been updated in line with all reviewer comments on the abstract as:<br>***Objectives*** *Social prescribing has provided a lifeline to people, capturing the importance of quality support offered via community connections. It has not been exempt from difficulties, resulting in low-quality evidence on effectiveness, in part due to high drop-out rates and lack of controls. While these have been the focus of recent research, less attention has been paid to the safety implications of social prescribing. This review aims to understand safety*

	implications of social prescribing interventions and to build a dark logic model of how harms are produced.<br>***Design*** *Using review of review (umbrella) methodology, we searched nine databases to June 2024.*<br>***Data Sources*** *Medline/Ovid, Embase, PsycINFO, Cochrane Library/Wiley, Web of Science, and Scopus) were searched from 1st January 2010 to 3rd June 2024.*<br>***Eligibility Criteria*** *Included reviews were systematic/ scoping/ narrative reviews or meta-analyses, which synthesised primary data collected from any person in receipt, involved in delivery, or the commissioning of social prescribing interventions. The context of the review was social prescribing interventions/services based in primary healthcare (statutory) or third sector (non-statutory) in any country. Only reviews that were published in English, and in peer-reviewed journals, were included.*<br>***Data Extraction and Synthesis*** *Two independent reviewers extracted data from included reviews using the Typology of Harms Framework (which includes five categories of harms associated with interventions: physical, psychological, group/social, equity and opportunity harms) and to build a dark logic model to understand what contributes to these harms.*<br>***Results*** *Sixteen reviews were included, reporting on social*

	prescribing research including a link worker. Of the identified harms, we found that opportunity harms (harm related to the cost, inappropriate or ineffective interventions) were most reported. There was also evidence extracted to suggest plausible psychological, equity (impact caused by inequity in provision, delivery, or access), and group/social harms (impact that overly or inadvertently excludes a person). No physical harms were identified.<br>***Conclusions*** *Social prescribing, as with any delivery of care, has the potential to cause harm. We identified a range of potential harms (psychological, group/social, equity, or opportunity harm) from social prescribing, however, it is unlikely to be an exhaustive list. We provide two clear outcomes: 1) the need for robust design of social prescribing research, including collection of data on the incidence and prevalence of harms, 2) recognition of the potential for harm from social prescribing and to address these where practicable.*
Introduction<br>o Was it the UK NHS or NHS in England – the reference seems to be NHS in England?<br>o Mentions dark logic – the authors need to explain what this is	Thank you this has been updated:<br><br>*National Health Service (NHS) in England*<br><br>Thank you this has been updated in the introduction to: *Dark logic models are frameworks that identify potential adverse or*

	unintended consequences of interventions, complementing traditional models focused on positive outcomes. Their application to social prescribing, allows for the mapping of risks such as negative outcomes (e.g., increased anxiety from pressure to engage), mechanisms (e.g., feelings of inadequacy), contextual factors (e.g., transport or financial barriers), and feedback loops (e.g., repeated disengagement reducing trust in providers).
Methods<br>o How was coding of data undertaken? By hand or some computer system?	Thank you this has been updated:<br><br>*All data were extracted using Microsoft Excel by hand, with custom data headings to collect data on review characteristics (country of origin, aims, design, synthesis method, sample size, co-morbidities, included references, and reported outcomes).*
Results<br>o Under psychological harms – is a needs assessment right for everyone? Could bring up issues that people don't want to discuss with a link worker and, therefore, to ask might cause harm. Link workers have talked about their dislike of using standardised measures because they are seen as impersonal and break the rapport they are trying to develop with patients. Any standardised needs assessment would need to be sensitive to this.<br>o I was not completely clear what opportunity constituted when written about in the results from line 260 onwards – perhaps this could be made clearer.<br>o There might be something about training and backgrounds of link worker and how this shapes how other professionals see this role. An individual link worker might come to be reflective, of all link workers to health professionals they interact with (for good or bad). But what they do as an individual may be shaped by their background and training (or lack thereof) as well as resource constraints and system level factor.<br>o I was surprised there was nothing about dependency on link workers –patients becoming dependent on them?<br>o Or nothing about burnout of link workers? Or of safeguarding risk	Thank you this has been updated:<br>- Needs assessment – thank you for your reflection. Our perceptive is that the needs assessment is an essential part of social prescribing and agree, it is a fundamental to person centred care. We have refined our wording of this point to reflect this: *Continuity was also affected by the lack of standardised needs assessment tools used by different services. The*

	absence of standardised needs assessments in social prescribing can result in inconsistent care because patient evaluations may vary significantly depending on individual link workers' judgment, experience, and available resources. Without a standardised approach, individual need may be overlooked or prioritised differently by different link workers (leading to inconsistent support). While standardised assessments are es essential for consistency and comparability, assessments must remain sufficiently flexible to accommodate individual circumstances, preferences, and cultural contexts. A rigid approach risks reducing person-centeredness and failing to address the psychology needs (leading to psychological harm).<br>- Opportunity harm and robust evidence – thank you this has been updated as follows to provide clarity: *Typically health focused interventions are evidence driven to*

	inform development, however there was a lack of improvements in patients post social prescribing.<br>- Training and backgrounds – Thank you for this. We have added a section to the discussion to reflect this: *There must also be consideration given to both the presentation and belief of the link worker role from other healthcare professionals, which could be reflective of the individuals training*.<br>- Link worker dependency and burn out: thank you for your reflection. Indeed this is something we were surprised to find little reference to in the included reviews.
• Discussion<br>o There seemed to be quite a lot of overlap between different types of harms – perhaps this could be alluded to in the discussion?<br>The authors used the Typology of Harms to help categorise data – it would be good to reflect on the strengths and limitations of having done so.<br>o The authors suggest a need for more creative ways to assess outcomes at the end of the discussion (line 397) – it would be good to have some thoughts on this and suggestions of what this might look like?<br>o In terms of limitations – the authors might reflect on this being a review of reviews so some relevant primary papers might not have been picked up a) because they were not included in the reviews that were included, or b) they were included in reviews but the sections on harms in these primary papers were not extracted into the reviews.	Thank you this has been updated:<br>- Overlap and typology: Thank you this has been updated in the limitation section of the discussion: *It must also be acknowledged the overlap between different types of harm within the Typology of Harms framework. While we applied clearly defined definitions of harm, the likelihood that harms exist in isolation is reductionist. We acknowledge that the*

	evidence available has been coded against certain definitions but there is future data required to establish the connectivity within the framework, and therefore a limitation of this review.<br>- Creative Assessment: Thank you, we have added: *(for example a visual self-assessment tool where patients rate their health across the three dimensions of health, social, physical, and mental).*<br>- Limitations: Thank you. We have added: *This review did not exclude reviews based on synthesis of outcomes of harms as it was known to the authors this has never been attempted in current social prescribing literature. While there was data to suggest harms, it is not an exhaustive review but provides a justification of why future research is needed. This*
• Tables<br>o Table 2 – I could not work out what the system was – usually articles in a table would be in alphabetical or chronological order – but I couldn't work out what system was used here.<br>o I feel there needed to be a table of included reviews – a table of characteristics of the reviews.	Thank you this has been updated:<br>- Thank you for your comments. We are un-sure which table is being referred to as there is only 1 table in the manuscript (definitions table). Supplementary materials 2 (now 3 following updates) is

	currently in order of references to correspond with the intext citations. We have checked each table to ensure consistent formatting and order of included reviews. We have also added a summary table of included review characteristics in the supplementary materials (2).
Reviewer 2	
Thank you for giving me the opportunity to review this article titled “The Potential Harms of Social Prescribing. A Global Umbrella Review and Dark Logic Model.” While the study is interesting and has the potential to fill an important knowledge gap, there are several aspects that make the manuscript fall short of that potential. Below are some comments for the authors to consider in light of this.	Thank you for taking the time to review our work. The feedback is very much appreciated and we hope we have updated our manuscript in line with suggestions.
**Overarching comments**<br>Rather than presenting evidence of actual harms as reported in the included reviews, the authors generally provide evidence on different factors that may contribute to different harms outlined in the typology of harms framework. For example, instead of providing evidence on widening of inequity (although vague what that means since the authors do not elaborate on the framework), they identify different conditions that could lead to “inequity” (unclear of what). While this would be fine and interesting, it requires more conceptual and theoretical work by the authors to properly reason and explain how and why the different conditions may result in different harms. One solution to this could be to structure the results around the dark logic model rather than thematically around the harms.<br>However, regardless of the focus chosen, the potential harms need to be more properly defined conceptually (e.g., what does psychological and equity harms mean?) and detailed empirically (i.e., what psychological or equity harms were identified in the literature, not just conditions that contribute broadly to “psychological harms”).<br>Overall, I have a hard time following the results since I do not have a clear understanding of the typology of harms framework after reading the manuscript. The authors should include a conceptual framework section where the typology of harms is presented and the five categories of adverse effects of interventions are properly defined and described. In addition, the equity and social/group harms seems conceptually very similar, so the authors should clarify how they differ. The text would also benefit from including	Thank you for your comments. We have redefined the framework and provided an updated table of evidence in the supplementary materials. The manuscript, table 1 has been updated to include application to social prescribing as an additional column.

some information on contexts/topics in which this framework has been previously used.	
1. Please define and describe what a dark logic model is when first mentioned. The different components of logic models should also be defined and the process of developing the dark logic model explained.	Thank you this has been updated in the introduction to: *Dark logic models are frameworks that identify potential adverse or unintended consequences of interventions, complementing traditional models focused on positive outcomes. Their application to social prescribing, allows for the mapping of risks such as negative outcomes (e.g., increased anxiety from pressure to engage), mechanisms (e.g., feelings of inadequacy), contextual factors (e.g., transport or financial barriers), and feedback loops (e.g., repeated disengagement reducing trust in providers).*
The umbrella review design should be described, and the authors should provide a rationale for conducting this specific type of review.	Thank you this has been updated in the methods to: . *Umbrella review methodology was necessary to investigate this research question as there are multiple systematic reviews and meta-analysis of social prescribing and an umbrella review enables the synthesis of high-level evidence across diverse interventions exposure. Additionally, there has been no single systematic review that is captured the potential harms of social prescribing instead made comment towards aspects of patient harm. Therefore, the volume of data required to answer the question is limited to those reviews that have extracted this data. Umbrella review methodology enhances*

	efficiency by leveraging existing system at reviews rather than duplicating efforts while following rigorous evidence synthesis guidelines, to set future research priorities. This review followed the Methodology for JBI Umbrella Reviews where appropriate.[ref]
The authors state that "no possible direct physical harms were reported in any of the reviews". Please explain what this means.	Thank you this has been updated for clarity to: *Direct physical harms were defined as 'Any adverse physical outcomes linked to prescribed activities (e.g., injury, health deterioration).' There were no data reported to suggest possible direct physical harms because of social prescribing in any of the reviews.*
The authors should provide some evidence for the statement "High drop-out rates caused by referral to inappropriate services and the lack of continuity of care once referred to services increase the risk of causing proximal psychological harm to patients". Alternatively, they should explain in more detail what this means, so the reader can understand the reasoning. What does high drop-out rates mean? Referral to inappropriate services and the lack of continuity of care? Proximal psychological harms?	Thank you this has been updated to provide an explanation of high-dropout rates and the impact to social prescribing as: *High drop-out rates refer to the proportion of patients who stop their involvement in an intervention (or in research) before its planned completion. In social prescribing practice, high drop-out rates can reduce the effectiveness of interventions, limit the achievement of intended health and wellbeing outcomes, and strain resources by requiring repeated engagement efforts. They may also act as a proxy for systemic barriers such as inadequate support, poor alignment with patient needs, or accessibility challenges, ultimately undermining trust*

	in the approach and its sustainability.
The paragraph on potential psychological harm is vague and somewhat speculative, providing neither tangible evidence nor a proper reasoning on the links between drop-out, lack of care continuity, lack of standardised needs assessment, and staff turnover to psychological harm (which is not properly explained). Hence, after reading this manuscript, I am not convinced about the potential psychological harms of social prescribing.	Thank you this has been updated for clarity: *Continuity was also affected by the lack of standardised needs assessment tools used by different services. The absence of standardised needs assessments in social prescribing can result in inconsistent care because patient evaluations may vary significantly depending on individual link workers' judgment, experience, and available resources. Without a standardised approach, individual need may be overlooked or prioritised differently by different link workers (leading to inconsistent support). While standardised assessments are es essential for consistency and comparability, assessments must remain sufficiently flexible to accommodate individual circumstances, preferences, and cultural contexts. A rigid approach risks reducing person-centeredness and failing to address the psychology needs (leading to psychological harm).*
The statement, "this created skill gaps in service provision, creating plausible proximal harm to patients", needs to be elaborated. Creating plausible proximal harm to patients, how? Why?	Thank you this has been updated for clarity to: *For example, differences in communication skills, knowledge of local resources, and ability to build rapport can result in inconsistent patient experiences and inequitable access to appropriate services. Patients assigned to highly skilled link workers*

	may receive comprehensive, tailored support, while others may experience fragmented care or limited options. This variability undermines the principle of equitable care and can distort the outcome of support, as improvements may reflect workforce capability rather than social prescribing intervention. This created plausible proximal harm to patients as they may receive support dependant on individual link worker competencies.
Similarly, the authors state that "seven reviews concluded there was a lack of training for social prescribers. [sic] leading to an inequity of provision" – how does lack of training lead to inequity of provision?	Thank you this has been updated as above.
Revise the sentence: "the framework was used to provide structure to the data extraction and provide guidance to data extraction".	Thank you this has been updated*: The framework was used to provide structure and guidance to the data extraction.*
Reviewer 3	
Thank you for the opportunity to review this manuscript, "The Potential Harms of Social Prescribing: A Global Umbrella Review." I am delighted to see a comprehensive review in this important and emerging field. This umbrella review addresses a critical knowledge gap, is well written, and effectively integrates dark logic modelling with the Typology of Harms Framework to enhance the interpretation of findings. The topic is highly relevant, and the paper's insights have the potential to significantly influence future research and inform the design of social prescribing (SP) interventions.<br>However, there are several areas that could be strengthened, particularly regarding the clarity of the methods, the presentation of results, and discrepancies between the protocol and this manuscript, which to my knowledge have not been amended in PROSPERO. I have outlined specific suggestions below, which I hope the authors find constructive.	Thank you for taking the time to review our work. The feedback is very much appreciated and we hope we have updated our manuscript in line with suggestions.
Abstract:<br>- The abstract is clear and well structured; however, it would benefit from mentioning the use of dark logic modelling within the methods.<br>- In the results section, please highlight that no physical harms were identified, this is an important and notable finding.<br>- Rather than defining the five harm categories in the abstract, focus on illustrative examples of actual findings within each (e.g. group	Thank you this has been updated in line with all reviewer comments on the abstract as:<br>***Objectives*** *Social prescribing has provided a lifeline to people, capturing the*

social harms such as age-based exclusion, language barriers, gender-specific groups, overly Westernised approaches).	*importance of quality support offered via community connections. It has not been exempt from difficulties, resulting in low-quality evidence on effectiveness, in part due to high drop-out rates and lack of controls. While these have been the focus of recent research, less attention has been paid to the safety implications of social prescribing. This review aims to understand safety implications of social prescribing interventions and to build a dark logic model of how harms are produced.* ***Design*** *Using review of review (umbrella) methodology, we searched nine databases to June 2024.* ***Data Sources*** *Medline/Ovid, Embase, PsycINFO, Cochrane Library/Wiley, Web of Science, and Scopus) were searched from 1st January 2010 to 3rd June 2024.* ***Eligibility Criteria*** *Included reviews were systematic/ scoping/ narrative reviews or meta-analyses, which synthesised primary data collected from any person in receipt, involved in delivery, or the commissioning of social prescribing interventions. The context of the review was social prescribing interventions/services based in primary healthcare (statutory) or third sector (non-statutory) in any country. Only reviews that were published in English, and in peer-reviewed journals, were included.* ***Data Extraction and Synthesis*** *Two independent reviewers extracted data from*

	included reviews using the Typology of Harms Framework (which includes five categories of harms associated with interventions: physical, psychological, group/social, equity and opportunity harms) and to build a dark logic model to understand what contributes to these harms.<br>***Results*** *Sixteen reviews were included, reporting on social prescribing research including a link worker. Of the identified harms, we found that opportunity harms (harm related to the cost, inappropriate or ineffective interventions) were most reported. There was also evidence extracted to suggest plausible psychological, equity (impact caused by inequity in provision, delivery, or access), and group/social harms (impact that overly or inadvertently excludes a person). No physical harms were identified.*<br>***Conclusions*** *Social prescribing, as with any delivery of care, has the potential to cause harm. We identified a range of potential harms (psychological, group/social, equity, or opportunity harm) from social prescribing, however, it is unlikely to be an exhaustive list. We provide two clear outcomes: 1) the need for robust design of social prescribing research, including collection of data on the incidence and prevalence of harms, 2) recognition of the potential for harm from social prescribing and to address*

	these where practicable.
Introduction:<br>- The introduction provides a strong overview and clearly defines SP. However, please include an early definition of "link worker" (thank you for noting that the role is known by various titles) . It is important to be explicit about how this term is defined for the purposes of the review as primary care vs third sector definitions vary.<br>- Some cited studies apply to specific patient groups only (e.g., the Cooper reference to mental health (line 64-66), the Skinner reference to Type 2 diabetes (line 67-70)). Please clarify this to avoid overgeneralisation.<br>- Consider expanding on how dark logic modelling is applied within the review. It is mentioned briefly in the introduction and conclusion, but it may warrant a dedicated paragraph in the methods describing what it is, how it was used, why it was chosen, and how it has been used in integration with the Typology of Harms framework. This is a strength of the review and deserves to be showcased.	Thank you this has been updated:<br>- Link worker definition – this has been added to the introduction as: *Social prescribing is delivered by a link worker, whose role is defined as a someone who connects an individual to support and services to improve their health (social, mental, physical) and wellbeing.*<br>- Specific patient groups – thank you this has been updated.<br>- Dark logic – thank you this has been added to the introduction: *Dark logic models are frameworks that identify potential adverse or unintended consequences of interventions, complementing traditional models focused on positive outcomes. Their application to social prescribing, allows for the mapping of risks such as negative outcomes (e.g., increased anxiety from pressure to engage), mechanisms (e.g., feelings of inadequacy), contextual factors (e.g., transport or financial barriers), and feedback loops*

	(e.g., repeated disengagement reducing trust in providers).
Methods:<br>- I note the protocol is published in PROSPERO which I have looked at.<br>- Did the authors consider including the preferred reporting items for overviews of reviews (PRIOR) statement to understand the trustworthiness and applicability of their findings. The PRIOR statement is endorsed by latest umbrella review guidelines (Fernandez et al. 2025 https://doi.org/10.1093/eurjcn/zvaf012)<br>- The inclusion criteria could be strengthened by outlining the PICO framework here, rather than only in the quality appraisal section.<br>- The justification for the 2010 start date (line 114) should be amended, as SP was referenced earlier (e.g., Friedli & Watson, 2004; South et al., 2008, doi:10.1017/S146342360800087X).<br>- Consider including an example search string, as terms are not currently outlined in the manuscript or PROSPERO record.<br>- The data extraction criteria described in the manuscript appear to differ from those in the PROSPERO protocol. Please clarify whether all variables listed in the protocol were extracted. If not, explain and justify any deviations.<br>- The explanation of the AMSTAR 2 tool is clear.<br>- The data synthesis section should indicate whether the planned categorisation (patient safety vs. healthcare system impact) was completed. If so, please report the findings accordingly.<br>- During synthesis, clarify whether quantitative and qualitative data were analysed separately or integrated, and justify the approach.<br>- Please explain how study overlap was managed - for example, whether a tool such as GROOVE (10.1002/jrsm.1557) was used.	Thank you this has been updated:<br>- PRIOR – Thank you for signposting this resource. We agree with the recommendation to consider the reporting item and feel we have met many of the criteria in our work. However we do not feel that applying these guidelines retrospectively would be aligned to the principles of the guide.<br>- Inclusion Criteria – Thank you we have updated this as follows: *Population: any person in receipt (patient, public, community member), involved in delivery (link worker or other title), or commissioning of social prescribing services (funders, governments) Intervention: social prescribing interventions/services (defined previously) based in primary healthcare (statutory care or first point of contract within a healthcare system) or third sector (non-statutory or non-governmental/ profit/ charity/ voluntary) in any country.*

	Outcome: any physical, mental, or social measure using qualitative, quantitative or mixed methods at an individual level.<br>- 2010 Start – Thank you, we have reworded this for clarity: *using analysis data from Web of Science and Scopus to track research publication trends for "social prescribing")*. We have also added two images of publication year data from each WoS and Scopus as evidence of this.<br>- Search String – Thank you we have added the following text for clarity in the methods: *For access to the search strategy please see published protocol.*<br>- Data Synthesis and PROSPERO- Thank you for this comment. We did indeed look to collect data aligned with the protocol, however there was many data categories in which there was no data reported. We have updated the manuscript accordingly to clarify this: *All data were extracted using Microsoft Excel by hand, with custom data headings to collect data on review characteristics (country of origin,*

	aims, design, synthesis method, sample size, co-morbidities, included references, and reported outcomes) as per protocol.<br>And<br>*In the protocol, it was stated data would be coded against either patient or healthcare system harm. However, this was not possible due to the limited data (quality and quantity) for healthcare system outcomes.*<br>- GROOVE - Thank you. In line with other reviewer comments we have used the GROOVE tool to identify overlap and have updated as follows:<br>Methods: *To assess the potential for overlap of reported data between included reviews, the GROOVE tool was used to calculate and interpret the overlap.*<br>Results: *Using the GROOVE tool to measure the overlap between primary studies reported by included reviews, indicated a slight overlap in interpretation. However, the corrected covered area was 3.5%, which is below the threshold of <5% of slight overlap (lowest category). For*

	GROOVE tool outputs please see supplementary materials 2.
Results:<br>- A flowchart of the literature search and study selection would be helpful (https://bmjmedicine.bmj.com/content/1/1/e000071)<br>- A summary table of study characteristics should be included in the main text or supplementary materials. It is mentioned as Supplementary Material 1, but not visible in the version provided. Also, on this point, regarding data extraction umbrella reviews "researchers should extract the number of eligible studies, the total sample size and (for binary outcomes) the number of events, the rationale for not performing a meta-analysis, and the descriptive conclusions" (https://bmjmedicine.bmj.com/content/1/1/e000071) – I'm not sure whether this was done or not because I cannot see the extraction table.<br>- The results are good and well-mapped to the Typology of Harm framework. However, it may be helpful for the reader if the results could be separated into patient safety, impact on those who deliver SP (link workers), and the impact on current health systems. This will help demonstrate which harms affect the specific population groups that are included in this umbrella review (patients, link workers)	Thank you this has been updated:<br>- Flowchart – Thank you, however we feel we have updated the methods section significantly with reviewer comments to address this. Please see substantial amendments and updates to the methods section.<br>- Summary table – Thank you for highlight this, we have ensured the supplementary materials table is on the system. We believe it may have been due to two files with the same name, which was our error.<br>- Results - Thank you for this comment. As previously stated, we hope to provide clarity with an update by adding: *In the protocol, it was stated data would be coded against either patient or healthcare system harm. However, this was not possible due to the limited data (quality and quantity) for healthcare system outcomes.*
Discussion<br>- The discussion could be strengthened by linking findings more explicitly to the complexity of SP as a multifaceted intervention.<br>- The protocol indicates that subgroup analyses (e.g., intervention	Thank you this has been updated:<br>- Linking to complexity: *It must also be*

types, pre–post outcomes) were planned. If these data were unavailable, please reflect on why this may be the case.<br>- Regarding the limitations, I would argue that the 2023 Muhl definition is established and adopted widely in the literature. The definition was coined through a rigorous Delphi study, it would be hard to justify the use of any other definition currently.<br>- Consider adding study overlap as a limitation	*acknowledged that social prescribing is a complex, multifaceted intervention that is fluid in delivery. The potential for harm is not static, and instead will be dependent on the individual, link worker, need level, design, delivery, and resource availability. This review provides the initial innovation in thinking to develop future thinking and how harm complexity can be reviewed.*<br>- Sub-group analysis: *In the protocol, it was stated data would be coded against either patient or healthcare system harm. However, this was not possible due to the limited data (quality and quantity) for healthcare system outcomes.*<br>- Limitations: Thank you for your reflection. We however feel that pre 2023 there were many definitions globally that were used and/or models of delivery. Indeed, Husk et al outline the four different models of social prescribing of which two did not include a link worker. (Husk K, Blockley K, Lovell R, Bethel A, Lang I, Byng R, Garside R. What approaches to social

	prescribing work, for whom, and in what circumstances? A realist review. Health & social care in the community. 2020 Mar;28(2):309-24.)<br>- Overlap: thank you. We had addressed overlap in a previous reviewer comment and applied the GROOVE tool to this work.
Reviewer 4	
Thank you for sharing this well-developed scoping review. I read it with great interest and even found myself exploring the concept of "dark logic models" further.	Thank you for taking the time to review our work. The feedback is very much appreciated and we hope we have updated our manuscript in line with suggestions.
However, several issues arose during my reading and review:<br>• The term dark logic is introduced but not clearly distinguished from general risk or harm frameworks. It remains unclear how this model offers additional explanatory value beyond the existing Typology of Harms Framework.	Thank you this has been updated to provide clarity:<br>*Dark logic models are frameworks that identify potential adverse or unintended consequences of interventions, complementing traditional models focused on positive outcomes. Their application to social prescribing, allows for the mapping of risks such as negative outcomes (e.g., increased anxiety from pressure to engage), mechanisms (e.g., feelings of inadequacy), contextual factors (e.g., transport or financial barriers), and feedback loops (e.g., repeated disengagement reducing trust in providers). Applying dark logic provides a starting point for the development, commissioning and delivery of social prescribing services to reflect upon how harms may arise in a clearly constructed framework.*

The model seems largely deductive and speculative. I found limited evidence in the included reviews to support the construction of causal chains of harm. Therefore, I recommend clarifying the theoretical contribution of the dark logic model: is it intended as a novel framework, an adaptation of existing models, or a visual summary?	Thank you this has been updated: *Applying dark logic provides a starting point (visual summary) for the development, commissioning and delivery of social prescribing services to reflect upon how harms may arise in a clearly constructed framework.*
Regarding the search strategy and the outcomes included, I offer the following observations:<br>• The absence of reported physical harms is interpreted as a "silence in the data," which feels speculative. It would be more robust to acknowledge the limitations of the included reviews in identifying such harms.	Thank you this has been updated*: Direct physical harms were defined as 'Any adverse physical outcomes linked to prescribed activities (e.g., injury, health deterioration).' There were no data reported to suggest possible direct physical harms because of social prescribing in any of the reviews.*
Psychological and opportunity harms are well described, but often lack quantitative data on prevalence or severity.	Thank you for your comment. We have significantly amended and updated this section of the results as follows:<br>*High drop-out rates, caused by referral to inappropriate services and the lack of continuity of care once referred to services, increased the risk of causing proximal psychological harm to patients. High drop-out rates refer to the proportion of patients who stopped their involvement in an intervention (or in research) before its planned completion. In social prescribing practice, high drop-out rates can reduce the effectiveness of interventions, limit the achievement of intended health and wellbeing outcomes, and strain resources by requiring repeated engagement efforts. They may also act as a proxy for systemic barriers such as inadequate support, poor alignment with patient needs or accessibility*

	challenges; ultimately, undermining trust in the approach and its sustainability. Three reviews reported on the high drop-out observed in social prescribing, which was typically measured between referral and entry to social prescribing, entry to social prescribing and initial appointment, or from initial appointment to onward support. [8 31 33] *Reviews reported a drop-out rate which ranged from 31% to 41% of patients enrolled on the intervention. The causes of the dropout were under-reported within the reviews and there was no evidence to suggest this area had been explored further.* [8 31 33] *However, one plausible contributing factor reported was the lack of continuity of care reported in three reviews.*[8 23 30] *High workforce turnover led to inconsistent care for patients, with continuity and trust being recognised as central to the success of social prescribing. Frequent changes in link workers disrupted the therapeutic relationship, which was commonly built on rapport and personalised understanding of a patient's social, emotional, and practical needs. This inconsistency could result in fragmented care plans, repeated assessments, and delays in connecting patients to appropriate community resources. Moreover, staff turnover could reduce institutional knowledge and weaken coordination between healthcare providers and community organisations; in turn, this is associated with diminished patient engagement and outcomes, as well as potential distal harm to patients due to inconsistency of support and how "participation in SP programs was disrupted by the high turnover of social prescribers".*[23]

	Continuity was also affected by the lack of standardised needs assessment tools used by different services. The absence of standardised needs assessments in social prescribing resulted in inconsistent care because patient evaluations may vary significantly depending on individual link workers' judgment, experience, and available resources. Without a standardised approach, individual need could be overlooked or prioritised differently by different link workers (leading to inconsistent support). While standardised assessments have been regarded as essential for consistency and comparability, assessments must remain sufficiently flexible to accommodate individual circumstances, preferences and cultural contexts. A rigid approach risks reducing person-centeredness and failing to address the psychology needs (leading to psychological harm). One review acknowledged the impact of COVID-19 on consistent delivery but also stated that "factors such as personnel changes… costly travel to referred activities, and referral delays resulted in a loss of engagement within social prescribing".[30]
The narrative synthesis is rich, but could be strengthened by more systematic coding or tabulation.	Thank you for your comment. We have updated the manuscript significantly based on reviewer feedback and hope this addresses your point.
Equity and group/social harms are discussed in terms of variability in service provision, yet the connection to actual patient outcomes appears weak. Many harms are inferred rather than directly reported.	Thank you this has been updated with clarity in line with other reviewer comments: *Social prescribers were identified to come from varied backgrounds and with*

	different levels of education.[35] *This led to some social prescribers having skills in social work, mental health, community development, or working with specific populations.*[28] *Furthermore, seven reviews concluded there was a lack of training for social prescribers. leading to an inequity of provision.*[8 23 26-30 35] *For example, differences in communication skills, knowledge of local resources, and ability to build rapport can result in inconsistent patient experiences and inequitable access to appropriate services. Patients assigned to highly skilled link workers may receive comprehensive, tailored support, while others may experience fragmented care or limited options. This variability undermines the principle of equitable care and can distort the outcome of support, as improvements may reflect workforce capability rather than social prescribing intervention. This created plausible proximal harm to patients as they may receive support dependant on individual link worker competencies.*
To enhance methodological transparency, I suggest clarifying how harms were extracted, coded, and synthesized. Additionally, consider including a supplementary table that maps each harm category to specific examples from the included reviews, with appropriate citations and contextual information.	Thank you this has been updated: *Umbrella review methodology was necessary to investigate this research question as there are multiple systematic reviews and meta-analysis of social prescribing and an umbrella review enables the synthesis of high-level evidence across diverse interventions*

	exposure. Additionally, there has been no single systematic review that is captured the potential harms of social prescribing instead made comment towards aspects of patient harm. Therefore, the volume of data required to answer the question is limited to those reviews that have extracted this data. Umbrella review methodology enhances efficiency by leveraging existing system at reviews rather than duplicating efforts while following rigorous evidence synthesis guidelines, to set future research priorities.

Scopus Publication Year Data Trend

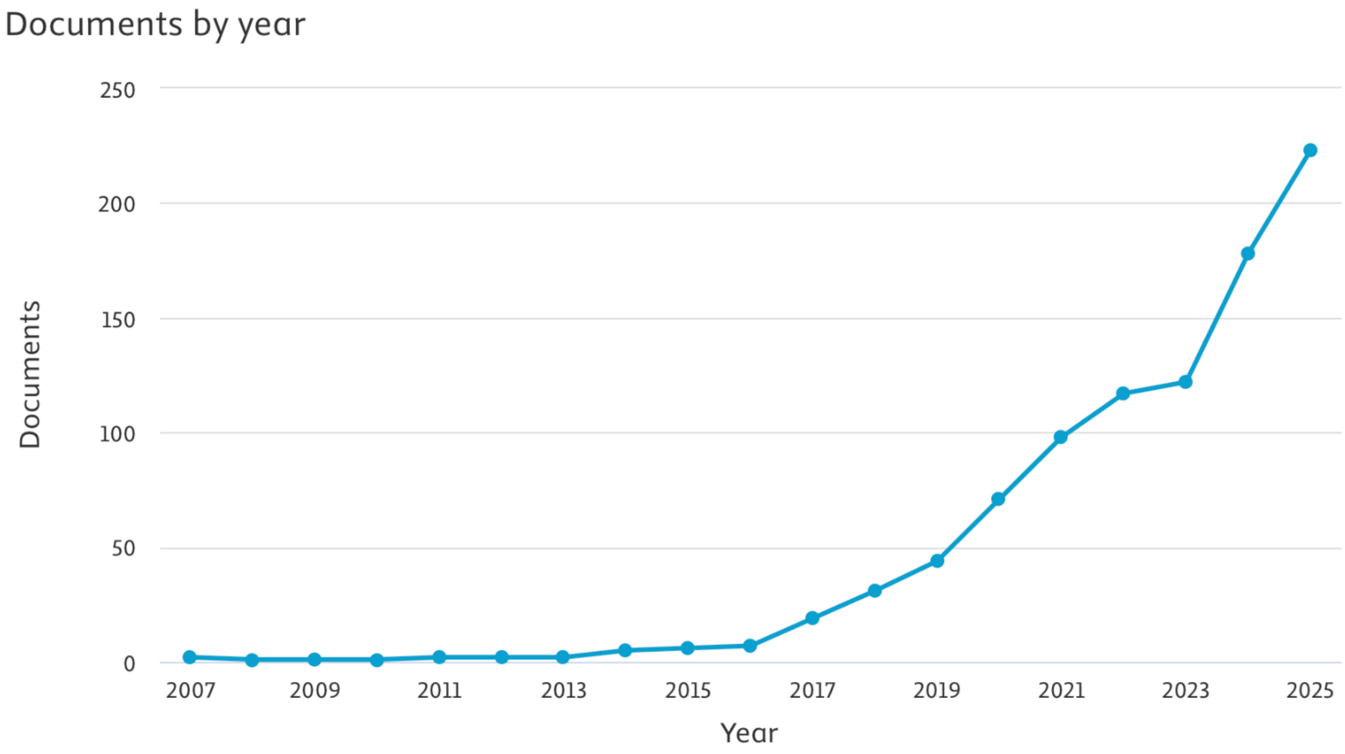

Web of Science Publication Year Data Trend

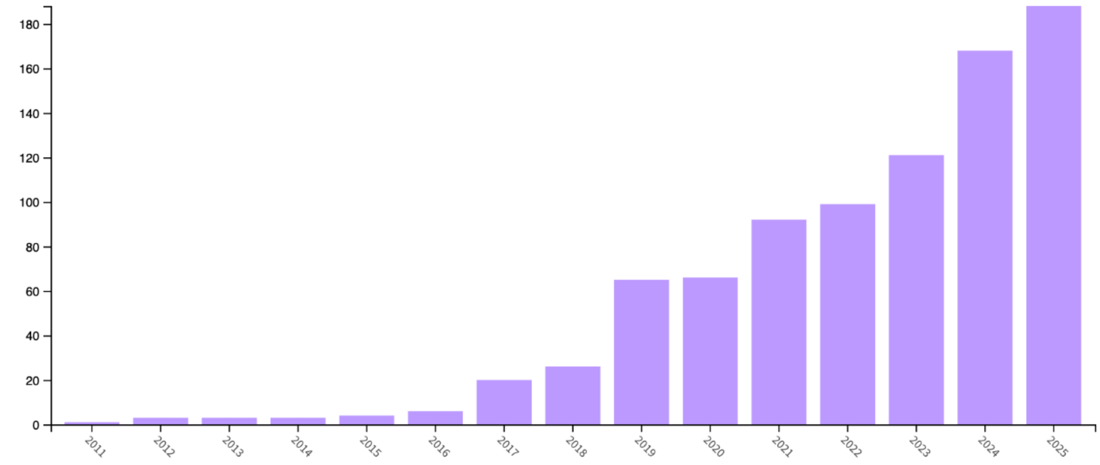

VERSION 2 - REVIEW

**Reviewer**	**1**
**Name**	**Tierney, Stephanie**
**Affiliation**	**University of Oxford, Nuffield Department of Primary Care Health Sciences**
**Date**	**09-Jan-2026**
**COI**	

I have looked through the responses to the comments made and I am happy with what has been proposed.

**Reviewer**	**2**
**Name**	**Jonsson, Frida**
**Affiliation**	**Umeå Universitet Medicinska fakulteten, Department of Social Work**
**Date**	**21-Jan-2026**
**COI**	

Thank you for the opportunity to review the revised version of "The Potential Harms of Social Prescribing: A Global Umbrella Review and Dark Logic Model." I would like to begin by commending the authors for tackling an important and underexplored aspect of social

prescribing. The focus on potential harms represents a timely contribution, particularly considering the growing implementation of such initiatives alongside an expanding yet still developing evidence base.

That said, I have several interrelated concerns regarding the conceptual framing, classification, and causal reasoning underpinning the identification of harms across the manuscript, which I do not feel have been sufficiently addressed in the revision considering my previous comments. In particular, there remain recurring ambiguities in how different types of harm (psychological, equity, and social/group harms) are analytically distinguished from one another, and how inferred risks and plausible mechanisms are differentiated from harms. In several instances, service level variability and other explanatory factors are treated as harms themselves, without clear evidence of corresponding outcomes at either individual or population levels. While these issues may indeed signal conditions under which harm could arise, the conceptual boundaries and causal links between risk factors, mechanisms, and outcomes remain insufficiently specified for the paper to make a valuable and valid contribution to the field.

Given the authors' aim of providing an initial framework to guide future thinking about harms in social prescribing, greater clarity in the analytical logic linking theoretical concepts to empirical observations is needed. Please consider the comments below as constructive suggestions for strengthening the conceptual coherence, internal consistency, and utility of the study.

1. The distinction between "equity harm" and "social harm" need to be clarified. As currently defined, both appear to capture overlapping processes of exclusion affecting particular groups. Equity harm is described as disparities in service access or delivery, while social harm is intended to capture exclusionary practices or stigmatization. In this light, the distinction remains unclear, as the latter may constitute a mechanism through which the former is produced. Without further specification, the categories are analytically difficult to distinguish and risk redundancy. The authors should therefore either sharpen the conceptual boundaries between these harms or explicitly acknowledge and theorize their overlap.

2. The classification and connection between high drop out rates and psychological harm is still insufficiently substantiated. The review does not report direct measurements of psychological outcomes such as emotional distress, anxiety, or feelings of isolation, nor does it provide evidence that individuals who dropped out experienced psychological harm. Furthermore, there is no synthesis of studies demonstrating a causal or temporal link between drop out and psychological outcomes, although the authors point to lack of continuity of care as a potential contributing factor. Instead, drop out appears to be treated as a proxy for psychological harm in itself, rather than as a system level failure, a disengagement outcome, or a risk factor that may contribute to psychological effects.

Against this backdrop, the authors have to specify which proximal psychological harms were directly identified in the reviewed studies and clarify how these harms are empirically and theoretically linked to high drop out rates.

3. A similar issue of conceptual ambiguity and weak causal specification arises in the treatment of equity harms. In this results section, equity harm is primarily inferred from variation in service delivery, workforce backgrounds, training, supervision, and appointment structures. While these findings clearly demonstrate implementation variability and service level risks, they do not in themselves constitute evidence of equity harm as defined by the authors, unless they are shown to produce systematic, group patterned disparities in access or delivery. As presented, variation in link worker competencies and intervention intensity is treated as equity harm rather than as a risk factor or mechanism through which inequities might arise. If the authors are to argue that social prescribing interventions produce equity harms, they need to specify which populations are disadvantaged and demonstrate how implementation variability leads to and is conceptually distinct from, observed inequitable outcomes.

Relatedly, findings currently classified as equity harms appear to align more closely with the authors' definition of social or group harms. The authors should therefore clarify their classification logic and either reassign such findings or provide evidence that these implementation variations produce systematic inequities for specific populations.

4. The authors need to include a reference to dark logic models.

VERSION 2 - AUTHOR RESPONSE

Reviewer Comments	Response
Reviewer: 1 Dr. Stephanie Tierney, University of Oxford	
I have looked through the responses to the comments made and I am happy with what has been proposed.	Thank you, Dr Tierney, for taking the time to review the amendments and approving this work.
Reviewer: 2 Dr. Frida Jonsson, Umeå Universitet Medicinska fakulteten	
Thank you for the opportunity to review the revised version of "The Potential Harms of Social Prescribing: A Global Umbrella Review and Dark Logic Model." I would like to begin by commending the authors for tackling an important and underexplored aspect of social prescribing. The focus on potential harms represents a timely contribution,	Thank you, Dr Jonsson, for taking the time to review this work in detail and for your comments on our approach. Please see a detailed response to each of your comments/ suggestions below.

particularly considering the growing implementation of such initiatives alongside an expanding yet still developing evidence base. That said, I have several interrelated concerns regarding the conceptual framing, classification, and causal reasoning underpinning the identification of harms across the manuscript, which I do not feel have been sufficiently addressed in the revision considering my previous comments. In particular, there remain recurring ambiguities in how different types of harm (psychological, equity, and social/group harms) are analytically distinguished from one another, and how inferred risks and plausible mechanisms are differentiated from harms. In several instances, service level variability and other explanatory factors are treated as harms themselves, without clear evidence of corresponding outcomes at either individual or population levels. While these issues may indeed signal conditions under which harm could arise, the conceptual boundaries and causal links between risk factors, mechanisms, and outcomes remain insufficiently specified for the paper to make a valuable and valid contribution to the field.	
Given the authors' aim of providing an initial framework to guide future thinking about harms in social prescribing, greater clarity in the analytical logic linking theoretical concepts to empirical observations is needed. Please consider the comments below as constructive suggestions for strengthening the conceptual coherence, internal consistency, and utility of the study.	Thank you for your comments. We have taken the time to go through each comment in detail and respond appropriately.
1. The distinction between "equity harm" and "social harm" need to be clarified. As currently defined, both appear to capture overlapping processes of exclusion affecting	Thank you for your suggestion we have updated the definitions of equity and social harm in Table 1 as follows:<br><br>*Equity Harm*<br>*Definition*

particular groups. Equity harm is described as disparities in service access or delivery, while social harm is intended to capture exclusionary practices or stigmatization. In this light, the distinction remains unclear, as the latter may constitute a mechanism through which the former is produced. Without further specification, the categories are analytically difficult to distinguish and risk redundancy. The authors should therefore either sharpen the conceptual boundaries between these harms or explicitly acknowledge and theorize their overlap.	*Any potential negative structural or systemic inequities in the design, provision, or delivery models of social prescribing that result in unequal access, quality of provision, or benefits specific groups based on characteristics (age, sex, gender, disability, culture).*<br>*Applied*<br>*Patterned disparities across population characteristics caused by institutional processes/ policy, allocation of resources, design of services, evidence used in decision making or organisational practices that systematically advantage some populations over others.*<br><br>*Social Harm*<br>*Definition*<br>*Any potential negative impact of interpersonal or community-level processes or beliefs (stigma, social stereotypes, scepticism or fear of certain people) that affect a person(s) ability to engage meaningfully with social prescribing or supporting organisation.*<br>*Applied*<br>*Harms caused by social dynamics (stigma, exclusionary behaviour, identity-based exclusion for example) that are engaged to cause disruption to relationships, discomfort, discouragement, or discontinued engagement in support.*
2. The classification and connection between high dropout rates and psychological harm is still insufficiently substantiated. The review does not report direct measurements of psychological outcomes such as emotional distress, anxiety, or feelings of isolation, nor does it provide evidence that individuals who dropped out experienced psychological harm. Furthermore, there is no synthesis of studies demonstrating a causal or temporal link between drop out and psychological outcomes, although the authors point to lack of continuity of care as a potential contributing factor. Instead, drop out appears to be treated as a proxy for psychological harm in itself, rather than as a system level failure, a disengagement outcome, or a risk factor that may contribute to psychological effects.<br>Against this backdrop, the authors have to specify which proximal psychological harms were directly identified in the reviewed studies and clarify how these harms are empirically and theoretically linked to high drop out rates.	Thank you for this comment. In relation to your comment around drop-out, our intention was not to present dropout as harm in itself, but rather as a well-established risk factor and indicator of vulnerability within health and social care settings. The literature outside our review already provides a strong evidential foundation for this connection, and we have clarified this more explicitly in the manuscript:<br><br>*Psychological Harm*<br>*Referral to inappropriate services and the lack of continuity of care once referred to services can increase drop-out rates amongst social prescribing participants.*<br><br>There is extensive empirical research demonstrating that disengagement, non-participation, and dropout are associated with adverse psychological outcomes, including reduced confidence, heightened anxiety, emotional distress, and perceived stigma. Pescheny et al. (2018) document that patients who disengage from social prescribing often report feelings of stigma and exclusion, both of which have recognised psychological consequences. These studies illustrate that the conceptual pathway from dropout to psychological harm is neither speculative nor novel but well-established within the broader evidence base. Second, the reviewer's comment assumes that a causal or temporal link must be demonstrated within the studies included in our review. However, our position is that dropout is widely understood within health services research as a proxy indicator of system-level failure and an established risk factor for subsequent psychological harm. This framing is consistent with long-standing conceptualisations of disengagement in the literature and, we feel, does not require more explicit causal sequencing within the individual studies we reviewed (practically also based on the limitations of the word count).

	Finally, we emphasise that the lack of continuity of care (of which dropout is a factor) is one of the best-documented patient safety problems, recognised for decades as contributing to emotional distress, uncertainty, and psychological burden. Discontinuity has been repeatedly linked to poorer outcomes and reduced patient trust across multiple contexts. Our review identifies dropout as a mechanism through which continuity is disrupted, and therefore as a pathway by which psychological harm may plausibly arise. This argument aligns with established patient-safety theory.
3. A similar issue of conceptual ambiguity and weak causal specification arises in the treatment of equity harms. In this results section, equity harm is primarily inferred from variation in service delivery, workforce backgrounds, training, supervision, and appointment structures. While these findings clearly demonstrate implementation variability and service level risks, they do not in themselves constitute evidence of equity harm as defined by the authors, unless they are shown to produce systematic, group patterned disparities in access or delivery. As presented, variation in link worker competencies and intervention intensity is treated as equity harm rather than as a risk factor or mechanism through which inequities might arise. If the authors are to argue that social prescribing interventions produce equity harms, they need to specify which populations are disadvantaged and demonstrate how implementation variability leads to and is conceptually distinct from, observed inequitable outcomes.	Thank you for your suggestion. We have updated the text as follows by adding clear notes of where the updated definition applies intext:<br><br>*Variation in how social prescribing services are delivered has been recognised as presenting possible equity harm. This stems from how services are designed (structural inequity in the design) but also includes the wide range of professional and non-professional backgrounds of social prescribers, a lack of or poor-quality training for new social prescribers, and/or high variability in leadership and supervision (systemic inequity in provision). Within the included reviews, it was commonly reported there was a wide range in the frequency of appointments that were offered.[25 28 30 35] Five reviews reported the length of intervention offered ranged from two months[25] to two years[30] , with others having no maximum length of support.[26 30] The total time available within appointments also varied, with some appointments lasting ten minutes and others lasting up to 45 minutes.[28 35]*<br>*Social prescribers were identified to have varied professional backgrounds and with different levels of education.[35] Consequentially, this led to some social prescribers having skills in social work, mental health, community development, or working with specific populations (patterned disparity based on equity of provision).[28] Furthermore, seven reviews concluded there was a lack of training for social prescribers, acknowledged as a risk of leading to an inequity of provision (systemic inequity).[8 23 26-30 35] For example, differences in communication skills, knowledge of local resources, and ability to build rapport could result in inconsistent patient experiences and inequitable access to appropriate services. Patients assigned to highly skilled link workers may receive comprehensive, tailored support, while others may experience fragmented care or limited options. This variability undermines the principle of equitable care and can distort the outcome of support, as improvements may reflect workforce capability rather than social prescribing intervention. This created plausible proximal harm to patients as they may receive support dependant on individual link worker competencies (inequity caused by institutional process and design of service).*<br>*Two reviews reported the wide variation in the length of training offered, reporting a range from a two-hour workshop to a 16-week course.[26 28] Not only did the*

	length of training differ, but reviews that included social prescribers' views concluded the training offered was inadequate (inequitable quality of provision).[23 26-28] *Gaps in training for link workers were reported as referral processes,*[23] *system navigation,*[26] *engaging with the third sector,*[27] *and managing complex patients.*[27] *Three reviews concluded that there was a lack of leadership and supervision offered to social prescribers,*[23 28 30] *which, along with being a possible equity harm, could also be a potential opportunity harm. Although not directly concluded, the lack of supervision could also be linked to the high levels of burnout among social prescribers.*
Relatedly, findings currently classified as equity harms appear to align more closely with the authors' definition of social or group harms. The authors should therefore clarify their classification logic and either reassign such findings or provide evidence that these implementation variations produce systematic inequities for specific populations.	Thank you for your comment. We believe to have address this in the responses to the above comments.

VERSION 3 - REVIEW

Reviewer 2

Name Jonsson, Frida

Affiliation Umeå Universitet Medicinska fakulteten, Department of Social Work

Date 23-Mar-2026

COI

The authors have adequately addressed my comments. Thank you.
